# Redox-metabolic cascade NIR-II prodrug for synchronizing compensatory HSPs/P-gp feedback with mitophagy flux impairment against intrinsic thermotolerance

**DOI:** 10.1016/j.ijpx.2026.100671

**Published:** 2026-09-26

**Authors:** Lan Cui, Weishuang Lou, Wencan Zhou, Mengyao Sun, Zhongchen Jiao, Mengdi Li, Tong Wang, Xinyu Liang, Xinmeng Jing, Shuoye Yang, Pengshuai Zhang, Lingbo Qu, Peng Li

**Affiliations:** a School of Biological Engineering, Henan University of Technology, Zhengzhou 450001, PR China; b School of Chemical Engineering, University of Birmingham, Birmingham B15 2TT, UK; c Institute for Complexity Science, Henan University of Technology, Zhengzhou 450001, PR China; d Institute of Chemistry, Henan Academy of Sciences, Zhengzhou 450046, PR China

**Keywords:** Nanoprodrug, NIR-II, Metabolic homeostasis, HSPs, Thermotolerance, Mitophagy flux

## Abstract

The intrinsic proteostasis of HSPs/P-gp remains formidable barriers in the multidrug resistance (MDR) and thermotolerance. To dismantle the compensatory HSPs and P-gp defense axis, we developed a metabolic reprogramming NIR-II nanoprodrug Lipid 2-DG@Au/DOX-ss, using gold liposome-nanorod loaded disulfide-doxorubicin prodrug DOX-ss and 2-deoxy-d-glucose (2-DG). Results revealed 2-DG dose-dependent metabolic perturbations of ATP, glucose, NAD^+^, LDH. Coupled with a 24.01-fold ROS enhancement, a 5.05-fold PINK1 surge, a 0.65-fold LC3-II accumulation, 0.29-fold p62 increase, S-phase arrest (up to 64%), intensive mitophagy flux impairment was initiated to terminal cell death via NF-κB/PINK1/LC3-II pathway. Proteomic analysis further elucidates the concurrent inhibition of HSP70 (A12A)/90/B1(27)-mediated protein folding with P-gp efflux pump downregulation and the disruption of mitochondrial autophagic clearance, as well as unveiling the potent in immune modulation, ferroptosis, and calcium homeostasis. This bioenergetics-derived nanoprodrug with HSPs/P-gp compensation and mitophagy impairment provides a precise approach for eradicating recalcitrant MDR malignancies.

## Graphical abstract

**Figure:**
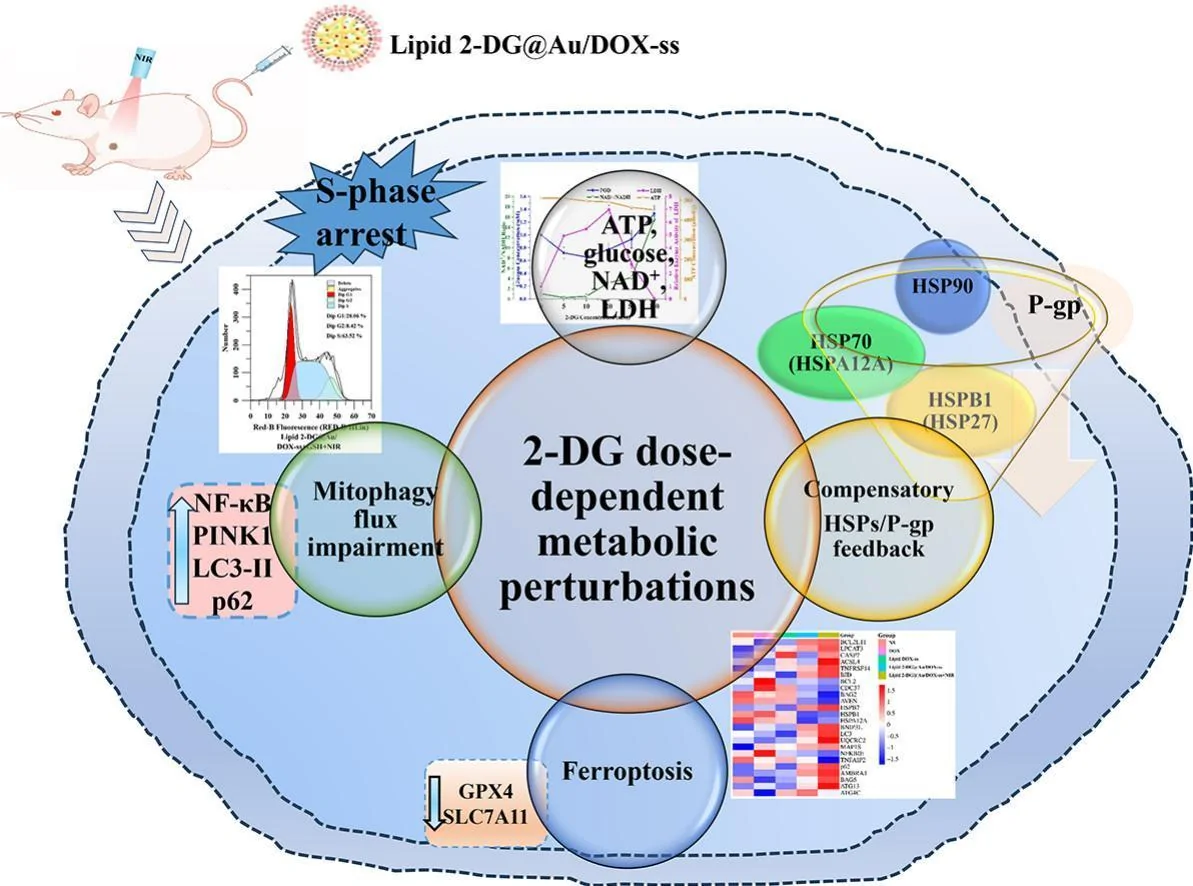

## 1 Introduction

Breast cancer currently ranks as a fifth malignancy due to the escalating morbidity and mortality rates (Abbott et al., 2026). The clinical pave of chemotherapy is frequently compromised by the systemic cardiotoxicity and P-glycoprotein (P-gp)-mediated multidrug resistance (MDR) (Liu et al., 2026). Liposomal encapsulation used to be a potential strategy to extend systemic circulation (Harter et al., 2026). However, the pharmacokinetics of hydrophilic drugs remains suboptimal at physiological temperatures due to the passive diffusion restriction from stable colloidal liposomes (Li et al., 2025). Dipalmitoyl phosphatidylcholine (DPPC)-based thermosensitive liposomes even fail to trigger drug penetration in the tumor core. Consequently, multidimensional platforms are essential to bypass P-gp barriers in chemo-resistant therapy (Zhou et al., 2025; Zou et al., 2026).

Second near-infrared (NIR-II, 900–1700 nm) irradiation has a superiority in non-invasive therapy via exceptional tissue penetration and minimal autofluorescence interference (Song et al., 2024; Tang et al., 2025). While tumoral ablation is often hindered by inherent non-specificity, low photothermal conversion efficiency, and collateral thermal injury to adjacent healthy tissues above 50 °C (Ding et al., 2025a). Critically, the cytoprotective heat shock proteins (HSPs) orchestrate cellular thermotolerance and aggravate resistance through sophisticated chaperone network (Shafeek et al., 2023; Wang et al., 2024). HSP27 (HSPB1) sequesters misfolded proteins and shuttled to HSP70 for refolding, then HSP90 facilitates the specific client proteins for maturation via intrinsic metabolism of glycolysis, tricarboxylic acid (TCA) cycle, and AMPK-mediated signaling. Compensatory cross-regulatory would be further reinforced through HSP90-mediated stabilization of HSF1 and synergistic interaction between HSP70 and HSP40/JDPs. HSP27 phosphorylation significantly modulates HSP70 recruitment as feedback loop (Rembialkowska et al., 2026). Diverse pharmacological agents have been explored but failed due to the compensatory upregulation among HSP family members (including HSP60, HSP70, HSP90, and HSPB1/HSP27), as well as the involvement of MHC-II-based suppressive immune responses (Ding et al., 2025c; Xu et al., 2024). Future research must focus on dismantling HSP family-derived compensatory circuits.

Metabolic reprogramming, such as glycolytic inhibitor 2-deoxy-d-glucose (2-DG), has demonstrated a pivotal role in suppressing the ATP-dependent chaperones like HSP90. However, its bioenergetic depletion-induced proteostatic collapse is rarely exploited in the compensatory feedback loops of HSPs family (Ding et al., 2025b). Based on our previous findings of cascade amplified oxidative stress via disulfide-modified doxorubicin prodrugs (DOX-ss) (Cui et al., 2024), we hypothesized that the synergistic integration of 2-DG with NIR-II photothermal redox prodrugs might trigger a stress cascade transition to reverse P-gp and HSPs compensatory loops for terminal mitophagy-dependent apoptosis.

In this study (scheme 1), a mild pH/GSH/NIR-II-responsive prodrugs lipid 2-DG@Au/DOX-ss was engineered via a seedless growth method and thermosensitive liposome assembly. Coupled with multifaceted redox stress and bioenergetic crisis, the compensatory loops of HSPA12A (HSP70), HSP90, and P-gp were severely broken, triggering an S-phase arrest and mitophagy flux impairment-induced apoptosis via NF-κB/PINK1/LC3 pathway. Our work elucidates a potent redox and bioenergetics-mediated prodrug to dismantle HSPs/P-gp compensatory circuits and mitophagy impairment for precision eradication of MDR cancers.

**Scheme 1:**
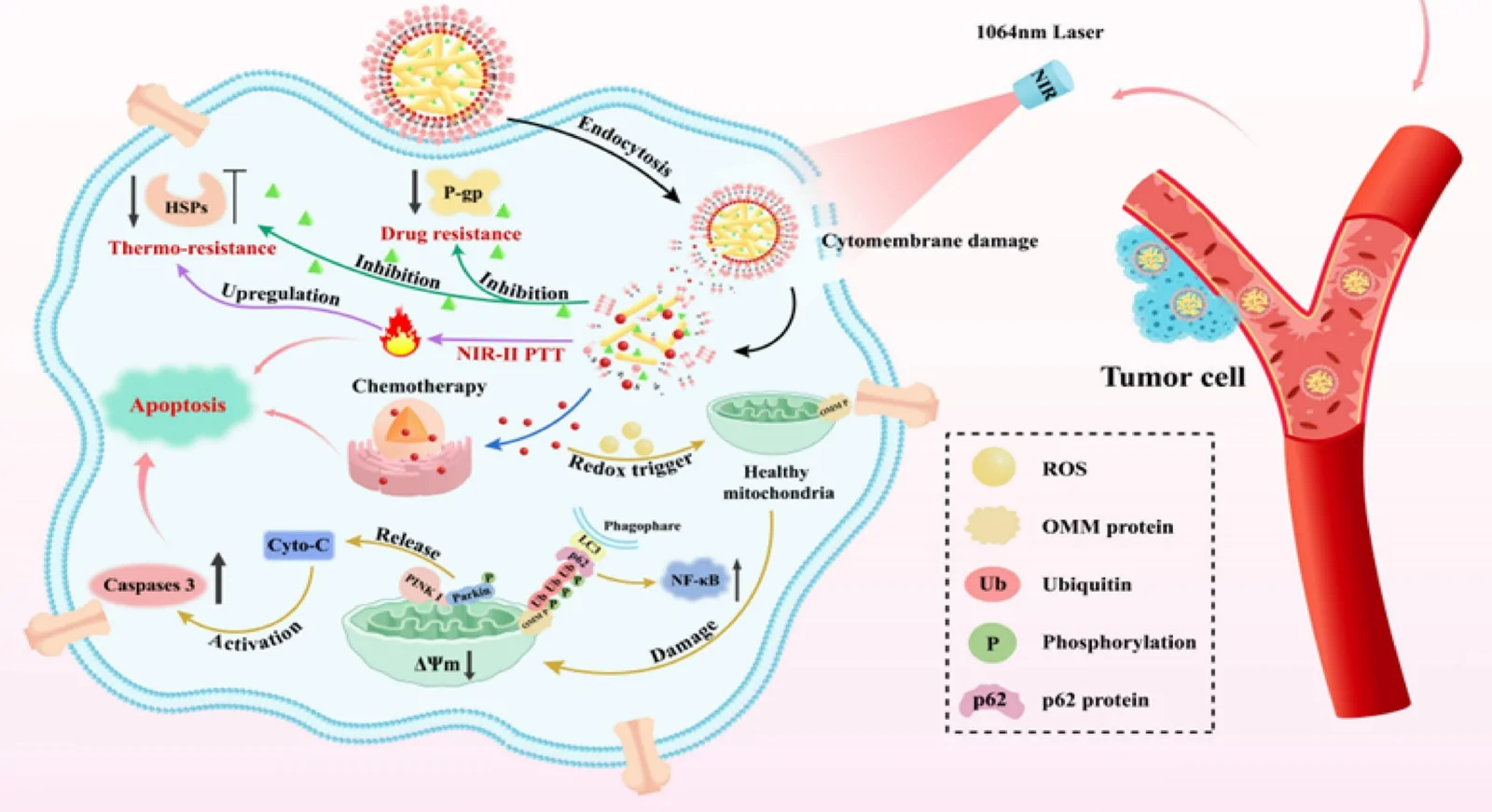
Schematic illustration of the construction of redox metabolic cascade prodrug and the mechanism of HSPs/P-gp compensation and mitophagy flux impairment-induced apoptosis via NF-κB/PINK1/LC3 pathway.

## 2 Materials and methods

### 2.1 Materials

Chloroauric trihydrate (HAuCl_4_·3H_2_O), hydroquinone, doxorubicin hydrochloride (DOX·HCl), Hexadecyltrimethylammonium bromide (CTAB), 4-dimethylaminopyridine, dichloromethane, triphosgene, cholesterol, 2-deoxy-d-glucose (2-DG) and reduced glutathione (GSH) were purchased from Shanghai Macklin Biochemical Co., Ltd. (Shanghai, China). 1,2-distearoyl-sn-glycero-3-phosphoethanolamine-N-[succinimidyl (polyethylene glycol)-2000] (DPSE-PEG 2000-NHS), 1,2-distearoyl-sn-glycero-3-phosphoethanolamine-N-[sulfydryl (polyethylene glycol)-2000] (DSPE-PEG 2000-SH) and L-alpha-Dipalmitoyl phosphatidylcholine (DPPC) were purchased from American Med. Chem. Express Biotechnology Co., Ltd. (Monmouth Junction, USA). Silver nitrate (AgNO_3_), hydrochloric acid (HCl) and sodium borohydride (NaBH_4_) were purchased from American Merck Drugs & Biotechnology Co., Ltd. (New Jersey, USA).

### 2.2 Cells culture and animals

MCF-7/ADR cells (catalogue number, BG006) were obtained from the Chinese Academy of Sciences (Shanghai, China). All cells were cultured in DMEM medium containing 10% fetal bovine serum under conditions of 37 °C and 5% CO_2_ atmosphere.

Female BALB/C nude mice (4 weeks old, about 16 ± 2 g, No.110011230106633484) were purchased from Beijing Vital River Laboratory Animal Technology Co., Ltd. (Beijing, China). The mice were housed in a specific pathogen-free environment at 20–25 °C and a humidity range of 50% - 60% and were exposed to a 12 h light/12 h dark cycles. The mice were fed with distilled water to drink and ordinary solid diet for one week prior to being used for experiment. Animal experiments were approved and performed following the animal protocol (license number, ZZU-LAC20241014[02]) in accordance with the guidelines of the Animal Center Committee of the National Drug Safety Evaluation Research Center of Zhengzhou University (Zhengzhou, China). 100 μL of MCF-7/ADR cells (5 × 10^6^ cells/mL) were injected subcutaneously into the right forelimb of mice to establish subcutaneous tumor models. Synergistic phototherapy was conducted when the tumor volume reached approximately 150 mm^3^.

The BALB/c mice bearing MCF-7/ADR tumor were intravenous injected with drugs (50 uL, DOX equivalent of 5 mg/kg) once every 3 days for five administrations. In the NIR group, the tumors were exposed to 1064 nm laser (0.5 W/cm^2^, 5 min) after 12 h post-injection. And the temperature change was observed using an infrared thermal camera for a total duration of 5 min of continuous laser irradiation. At the end of 15 days of treatment, mice were sacrificed by cervical dislocation after 1.3 μL of avertin anesthesia per milligram of mouse.

### 2.3 Synthesis of NIR-II-responsive Au NRs

Au NRs was synthesized by the seedless growth method using hydroquinone as reducing agent. CTAB (0.1 M,10 mL) solution was added to a round-bottom flask and sonicated until clear and transparent. Subsequently, HAuCl_4_ (0.01 M, 400 μL), AgNO_3_ (0.1 M, 23 μL), HCl (1 M, 10 μL) and hydroquinone (0.1 M, 540 μL) aqueous solution were added in sequence under stirring at 400 rpm for 15 min, then the color of solution changed from orange to transparent and colorless. Subsequently, NaBH_4_ (0.01 M, 10 μL) ice-cold solution was added to the above growth solution, and stirred for 15 s at 400 rpm, and then extended the mixture reaction and aging time to 24 h at 25 °C. Finally, the Au NRs (the precipitate) was obtained by twice centrifugation at 12000 rpm for 40 min at 25 °C. Then the resulting Au NRs were resuspended in ultrapure water (1 mL) and stored at 4 °C for further study.

The effects of the addition of NaBH_4_, AgNO_3_, and HCl on the synthesis of NIR-II responsive Au NRs were respectively investigated.

### 2.4 Synthesis of Lipid DOX-ss

DOX-ss was synthesized according to our previous methods.(Cui et al., 2024) DOX·HCl (58 mg) and 4-dimethylaminopyridine (48 mg) were dissolved in dichloromethane (20 mL). Then triphosgene (274 mg) was added under N_2_ environment and stirred at 25 °C for 30 min. Subsequently, 2-((2hydroxyethyl) disulfanyl) methacrylate (20 μL) was added to the above mixture and stirred at 25 °C for 24 h. DOX-ss was obtained after drying with anhydrous magnesium sulfate, filtration and rotary evaporation.

Synthesis of Lipid DOX-ss: DSPE-PEG2000-NHS (67 mg) and DOX-ss (20 mg) were dispersed in DMSO (10 mL), respectively. Then triethylamine (12 μL) was added under stirring at 600 rpm, 25 °C for 24 h. The product Lipid DOX-ss was obtained after dialysis (molecular weight cut-off of 2000 Da) for 48 h and freeze-drying for 12 h under vacuum level 200 mTorr.

### 2.5 Synthesis of Lipid 2-DG@Au/DOX-ss

Lipid DOX-ss (50 mg), DSPE-PEG2000-SH (18.5 mg), DPPC (25 mg) and cholesterol (12.5 mg) were suspended in 10 mL of chloroform/methanol mixture (molecular ratio of chloroform and methanol was 3:1), then rotary evaporated to form a film at 40 °C, 100 r/min for 30 min and dried in vacuum drying oven at 40 °C for 12 h. Then, 18 mL Au NRs (0.1 mg/mL) and 2-DG (5 mM) aqueous solution were added to round-bottom flask and then sonicated in an ice bath for 3 min. The Lipid 2-DG@Au/DOX-ss were purified by centrifugation at 12,000 rpm for 15 min. After that, the resulting sediment was resuspended in ultrapure water (1 mL) and stored at 4 °C for further study.

### 2.6 Photothermal performance of NIR-II prodrug

The photothermal conversion of NIR-II prodrug was investigated under NIR irradiation within 5 min at 1 W/cm^2^, respectively. The photothermal conversion efficiencies were calculated according to Eqs. (1), (2), (3), (4). The curve was plotted with *t* as y-axis and *−* *lnθ* as x-axis, respectively. Then the system thermal transfer time constant (*τ*_*s*_) was calculated by fitting the slope of straight line to determine *hS*. Subsequently, the energy generated with an equal amount of pure water (*Q*_*0*_) was calculated. Finally, the photothermal conversion efficiency (*ŋ*) was calculated according to the value of *hS* and *Q*_*0*_.

(1) $$\theta =\frac{T-T_{0}}{T_{\max}-T_{0}}$$

(2) $$\mathrm{hS}=\frac{\Sigma_{i}m_{i}C_{p,i}}{\tau_{s}}$$

(3) $$Q_{0}=\frac{\text{cmΔT}}{t}$$

(4) $$ŋ=\frac{\mathrm{hS}\left(T_{\max}-T_{0}\right)-Q_{0}}{I\left(1-10^{-A}\right)}$$

*T, T*_*max*_ and *T*_*0*_ represent temperatures of solutions at different moments under natural cooling conditions, the highest temperature when reached equilibrium under laser irradiation, and the initial temperature, respectively. *h, S, m*_*i*_ and *C*_*p,i*_ represent the heat transfer coefficient, surface area of container, the weight and specific heat capacity of components, respectively. *c, m ΔT* and *t* represent the specific heat capacity, mass temperature decrease value, and heat dissipation time of pure water after laser turning off, respectively. *A* and *I* represent the absorbance of nanomaterials and the power of laser, respectively.

### 2.7 In vitro drug release

Drug release of Lipid 2-DG@Au/DOX-ss were investigated under pH, NIR and GSH conditions. The NIR group was exposed to a 1064 nm laser at 1 W/cm^2^ for 5 min at regular intervals. 3 mg sample was dispersed in 3 mL of corresponding PBS solution under the specified conditions, then placed in a dialysis bag with a molecular weight cut-off of 500 Da under continuous shaken at a 37 °C corresponding PBS solution (30 mL). 3 mL of release fluid was withdrawn at regular intervals, meanwhile replenished with 3 mL fresh PBS with appropriate conditions. The absorbance at 480 nm was measured to calculate the cumulative release rate of DOX according to the standard curve of DOX at different conditions. Then the cumulative release rate was plotted with time as the horizontal coordinate and release rate as the vertical coordinate.

### 2.8 In vitro cytotoxicity

Normal human hepatocytes (*L*−02) and multidrug-resistant breast cancer cells (MCF-7/ADR) in logarithmic growth phase were collected, digested, counted and seeded in 96-well plates at a density of 10^4^ cells per well. The old medium was removed after incubation at 37 °C for 12 h. Empty and drug-loaded nanoparticles were added and incubated at 37 °C for 24 h and 48 h, respectively. Next, 100 μL MTT (20%) was added to the 96-well plate at 37 °C for 4 h incubation. The absorbance was measured at 490 nm after shaking for 10 min and the cell survival rate was calculated according to Eq. (5) compared with the blank control.

(5) $$\mathrm{Cell viability}\;\left(\%\right)=\left(A_{\mathrm{sample}}-A_{\mathrm{control}}\right)/\left(A_{\mathrm{cell}}-A_{\mathrm{control}}\right)\times 100\%$$

*A*_*sample*_, *A*_*control*_, *A*_*cell*_: the absorbance of cells incubated with nanomedicine, medium and without treatment at 490 nm.

### 2.9 Hemolytic experiments

5 mL of mouse blood was centrifuged at 1000 rpm for 5 min under 4 °C, then rinsed with saline to make the supernatant no longer appear red color. Subsequently, RBC was prepared into a 2% cell suspension via saline and transferred to 1.5 mL centrifugal tubes for hemolytic analysis. Then 0.25 mL Saline (negative control: 0% hemolysis), 1% TritonX-100 (positive control, 100% hemolysis) and nanoparticles were added to the above centrifugal tubes and incubated at 37 °C for 2 h. After centrifugation at 3000 rpm for 5 min, 100 μL supernatant was taken into a 96-well plate to measure the absorbance at 540 nm. Finally, the hemolysis rate was calculated to evaluate the biosafety of nanoparticles during blood circulation according to Eq. (6).

(6) $$\mathrm{Hemolytic Rate}\left(\%\right)=\left(A_{\mathrm{sample}–}\;A_{\mathrm{negative control}}\right)/\left(A_{\mathrm{positive control}}-A_{\mathrm{negative control}}\right)\times 100\%$$

*A*_*sample*_, *A*_*negative control*_ and *A*_*positive control*_: the absorbance of the experimental group, the negative control group and the positive control group at 540 nm.

### 2.10 Cell membrane integrity analysis

MCF-7/ADR cells were distributed in 96-well plates with a density of 10^4^ cells per well and cultured for 12 h. Drug-loaded nanoparticles were added and cultured for 4 h and 24 h after removing the old medium. Then, 80 μL of the supernatant was aspirated and 60 μL of the LDH working liquid were added separately. Next, the mixture was then incubated in the dark at 25 °C for 30 min. Lastly, the absorbance at 490 nm was measured by microplate reader.

### 2.11 Endocytosis analysis

MCF-7/ADR cells were inoculated in 6-well plates with a density of 10^5^ cells per well and cultured for 12 h. Drug-loaded nanoparticles were added and cultured for 4 h and 24 h after removing the old medium. The cells were washed with PBS twice and incubated with Hoechst 33342 for 15 min away from light. Subsequently, each well was fixed with 4% cell tissue fixative for 20 min after washing with PBS, then observed by fluorescence microscope.

### 2.12 Detection of ROS intensity

MCF-7/ADR cells were inoculated in 6-well plates. After removing the old medium, drug-loaded nanoparticles were added and kept for 4 h and 24 h culturation. Then intracellular reactive oxygen indicator DCFH-DA was added in the dark and incubated at 37 °C for 20 min. After fixing with 4% cell tissue fixative for 20 min, the ROS fluorescence intensity was observed through fluorescence microscopy.

### 2.13 Measurement of cellular ATP levels

MCF-7/ADR cells were respectively inoculated at a density of 10^5^ cells per well and then incubated for 24 h. Cells were then treated with different groups for 24 h. Then, the cells were washed with PBS twice, and harvested with lysate at 4 °C for 1 min. After centrifugation, ATP bioluminescent assay kit (Sigma-Aldrich) was used to assess the intracellular ATP levels.

### 2.14 Expression of HSP70/90 and P-gp through western blot

MCF-7/ADR cells were inoculated in 6-well plates. Then, drug-loaded nanoparticles were added and cultured for 24 h. As another control group, the HSPs inhibitor-(−)-epigallocatechin gallate (EGCG, 50 μM) and Lipid 2-DG@Au/DOX-ss were mixed and incubated to investigate the inhibitory effect of Lipid 2-DG@Au/DOX-ss on HSP70/90. After the cell lysis on ice for 15 min, the protein samples were collected, heat in metal bath at 37 °C for 30 min and separated by SDS-PAGE gel electrophoresis. Then, the protein was transferred to polyvinylidene difluoride membrane via wet transfer method. After treating the primary antibody of the target protein, the unbound antibody was removed through rinsing, and then the membrane was processed with the corresponding secondary antibody for enzyme immunolocalization. Finally, the gray values of protein were analyzed and quantified by the Image J software (Fiji).

### 2.15 Detection of mitochondrial membrane potential

MCF-7/ADR cells were inoculated in 6-well plates and cultured for 12 h. Next, the drug-loaded nanoparticles were added and cultured for 4 h and 24 h after removing the old medium. The cells were processed with Rhodamine 123 staining solution for 20 min under dark conditions. Finally, the fluorescence intensity was observed by fluorescence microscopy and quantified by the Image J software.

### 2.16 Subcellular localization analysis of lipid 2-DG@Au/DOX-ss

MCF-7/ADR cells were seeded at a density of 10^6^ cells per well in petri dishes and incubated for 24 h. Then, Lipid 2-DG@Au/DOX-ss was added to each well and incubated at 37 °C for 30 min, 3 h, 24 h and 48 h, respectively. Then the old medium was removed and washed twice with PBS to remove the residual nanoparticles. Cells were subsequently fixed with 2.5% glutaraldehyde at 4 °C for 24 h, fixed with PBS solution containing 1% OsO4 for 1 h, and then dehydrated in a stepwise series of ethanol, acetone, and propylene oxide and embedded in epoxy resin. The slices were stained with uranyl acetate and lead citrate and finally observed by transmission electron microscope.

### 2.17 Cell cycle arrest analysis

MCF-7/ADR cells were seeded in petri dishes at a density of 10^6^ cells per well and incubated for 24 h. Then, the drug-loaded nanoparticles were added and cultured for 12 h and 24 h. After incubation, the cells were collected in EP tubes and washed several times with PBS, meanwhile pre-cooled 70% ethanol was added dropwise and fixed at 4 °C overnight, then the cells were treated with PI staining solution for 30 min in dark to identify the cell cycle arrest by flow cytometry.

### 2.18 Apoptosis analysis

MCF-7/ADR cells were seeded in petri dishes at a density of 10^6^ cells per well and incubated for 24 h. Then, the drug-loaded nanoparticles were added and cultured for 12 h and 24 h. After cultivation, the cells were collected in EP tubes and washed several times with PBS solution, then the cells were mixed with Annexin *V*-FITC and processed with PI staining solution for 10 min to identify the apoptotic cells by flow cytometry.

### 2.19 In vivo antitumor activity

When the tumor volume reached approximately 150 mm^3^, the mice were randomly divided into five groups (*n* = 5): saline (NS), DOX alone, Lipid DOX-ss, Lipid 2-DG@Au/DOX-ss, Lipid 2-DG@Au/DOX-ss under NIR laser stimulation. The drugs were injected intravenously once every 3 days (DOX equivalent, 5 mg/kg). In the NIR group, the tumors were irradiated with a 1064 nm laser at a power of 0.5 W/cm^2^ for 5 min after the drug administration for 12 h, and the temperature change was observed using an infrared thermal imager. The body weight and tumor volumes were recorded and plotted before each administration, and tumor volumes were calculated according to Eq. (7).

(7) $$V=\frac{a\times b^{2}}{2}$$

*V, a and b*: tumor volume, tumor length, tumor width.

At the end of the experiment, the mice were anesthetized for blood sampling and routine blood analysis. The serum biochemistry indicators including alanine aminotransferase (ALT), aspartate aminotransferase (AST), alkaline phosphatase (ALP), blood urea nitrogen (BUN) and uric acid (UA) were detected. Meanwhile, the vital organs (heart, liver, spleen, lung and kidney) of mice were harvested and pathologically analyzed using H&E staining to evaluate the biosafety of the drug-loaded nanoparticles. Then, the tumors were harvested for weighing, immunohistochemical staining and immunofluorescence staining using H&E, TUNEL, Cyto-C, HSP70, HSP90, CD 31, Ki 67, Caspase 3 staining using the microscope.

### 2.20 Proteomics analysis

At the end of the experiment, the tumor tissues were harvested, washed with pre-cooled saline and sheared into small pieces of 0.5 cm. The above tissues were loaded into pre-cooled cryopreservation tubes, quick-frozen in liquid nitrogen for more than 5 min and transferred to −80 °C refrigerator for freezing. Sequencing libraries were created using Thermos Scientific Perce BCA Kit. Based on the quantitative results of the peptides, the peptides were analyzed using VanquishNeo device in combination with the timsTOF Ultra2 Mass Spectrometer (Bruker, Germany) at Majorbio Bio-Pharmaceutical Technology Co. Ltd. (Shanghai, China).

The data were analyzed through Majorbio Cloud (https://cloud.majorbio.com/). The differentially expressed proteins were identified by the thresholds of fold change (>1.2 or < 0.83) and *P*-value <0.05. All the identified proteins were functionally annotated using GO (http://geneontology.org/) and KEGG pathways (http://www.genome.jp/kegg/). Further, GO and KEGG enrichment analyses were conducted using these differentially expressed proteins. Protein-protein interaction analysis was performed using String v11.5.

### 2.21 Statistical analysis

All statistical data were presented as mean ± standard deviation of at least three independent measurements. Statistical analyses were performed using Origin 2018 software, Image J (version 1.8.0) and GraphPad Prism software (version 8.0). An unpaired two-tailed Student's *t*-test and a one-way ANOVA test analysis were employed to assess the statistical differences. Sample size (*n*) and probability (*P*) value for each statistical analysis were indicated in the corresponding figure legend. *, **, *** and **** in the figures were considered statistically significant and extremely statistically significant at *p* < 0.05, *p* < 0.01, *p* < 0.001 and *p* < 0.0001, respectively.

## 3 Results and discussion

### 3.1 Construction and characterization of Lipid 2-DG@Au/DOX-ss

Au NRs were synthesized via a seed-mediated growth method, with the parameters of NaBH_4_, HCl, and AgNO_3_ concentrations systematically optimized for size, morphology, and NIR-II absorbance (Fig. S1A-B). Specifically, an excess of NaBH_4_ promoted Au NRs aggregation but gradually assumed spherical particles and tended to be uneven, which may be preferable to the strong reducibility of NaBH_4_ in Au nucleus growth during LaMer burst (Zhang et al., 2026b). In contrast, the addition of HCl significantly enhanced the aspect ratio and uniformity of Au NRs by inhibiting the anisotropic overgrowth of the seeds (Mayaka and Alocilja, 2025). Furthermore, increasing AgNO_3_ concentrations moderately enhanced the aspect ratio, which was ascribed to the relative weak ionic strength effect on radial Au NRs deposition. TEM images confirmed that Au NRs maintained an average diameter (length 92.3 ± 11 nm, width 11.6 ± 3 nm), high length-to-diameter ratio (7.96 ± 0.30) and absorption peak in the NIR-II region (1088 nm) at the optimal parameters (HAuCl_4_: 400 μL, 0.01 M, AgNO_3_: 23 μL, 0.1 M, HCl: 10 μL, 1 M, hydroquinone: 540 μL, 0.1 M, CTAB:10 mL, 0.1 M, NaBH_4_: 10 μL, 0.01 M). The X-ray diffraction analysis (Fig. S1C) further revealed the face-centered cubic structure of Au NRs at 2θ angles of 38.54°, 44.50°, 64.94°, and 77.74°, corresponding to the 111, 200, 220, and 311 facets of Au NRs, respectively (Liu et al., 2024; Zhang et al., 2026a).

As shown in Fig. 1A-D, the simultaneous presence of Au, N, S, and P elements demonstrated the notable ligand modification of Lipid 2-DG@Au/DOX-ss. Moreover, the negatively charged liposomes promoted the zeta potential of Lipid 2-DG@Au/DOX-ss shift from 31.4 ± 2.42 mV to −14.9 ± 0.35 mV, further confirming the existence of liposomes for favorable endocytosis. The UV absorbance spectra (Fig. 1E) revealed surface plasmon resonance peaks of Au NRs and Lipid 2-DG@Au/DOX-ss at 1088 nm and 1068 nm, respectively, indicating that the modification did not significantly affect the optical characteristics of Au NRs.

**Fig. 1:**
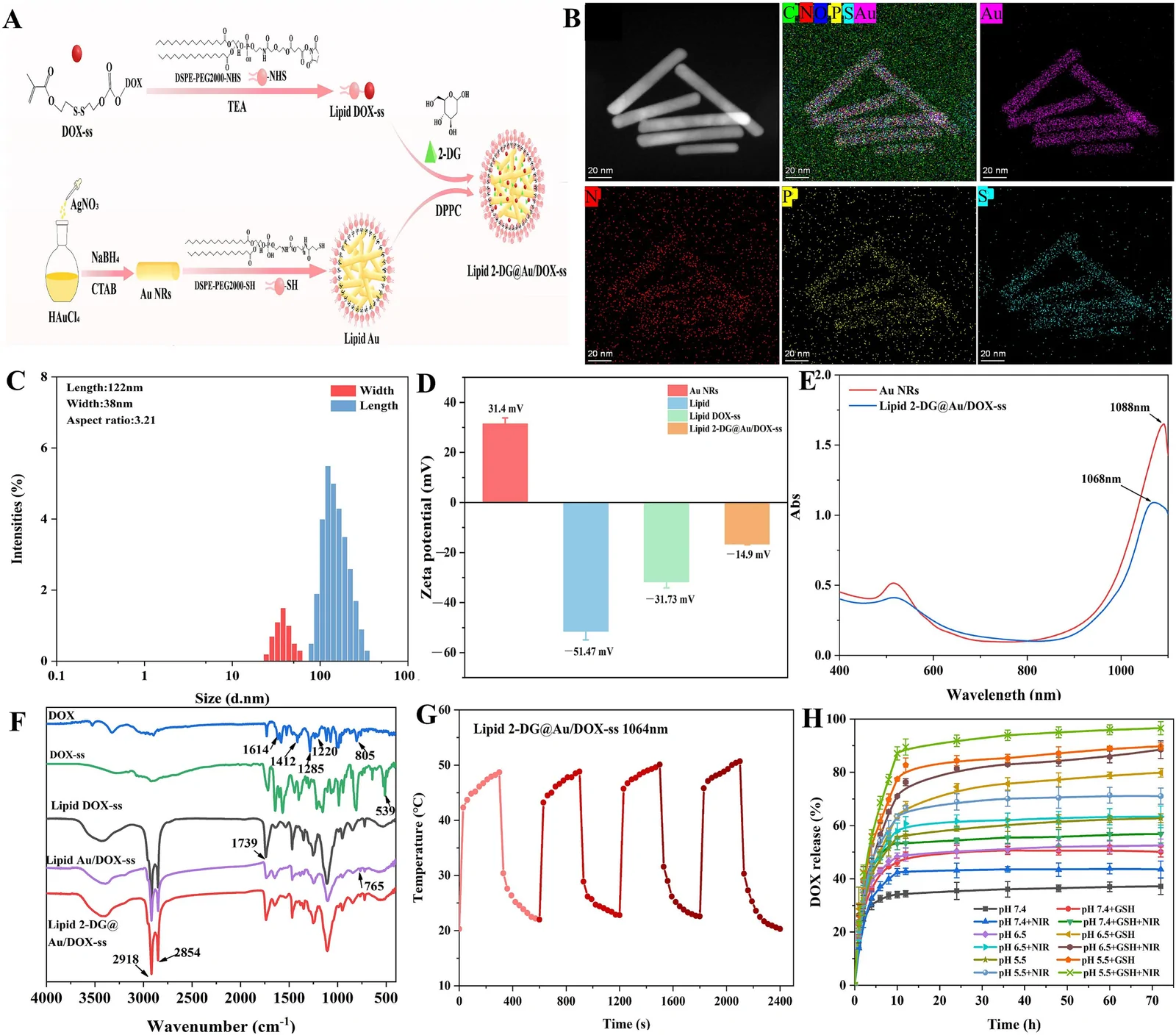
Preparation and characterization of Lipid 2-DG@Au/DOX-ss. (A) Schematic illustration of the synthesis of NIR-II-responsive nanoreactor. (B) Representative TEM images and elemental mapping of Lipid 2-DG@Au/DOX-ss. Scale bars: 20 nm. (C) Hydrodynamic size distribution of Lipid 2-DG@Au/DOX-ss. (D) Zeta potential of Au NRs, Lipid, Lipid DOX-ss and Lipid 2-DG@Au/DOX-ss. *n* = 3 independent experiments. (E) Determination of surface plasmon resonance absorption peaks by UV–Vis-NIR spectra. (F) FT-IR spectrum. (G) Photothermal conversion curves of Lipid 2-DG@Au/DOX-ss over four laser irradiation on/off cycles (1064 nm, 1 W/cm^2^, 5 min). (H) pH/GSH/NIR-II-responsive release profiles (pH: 5.5, 6.5 and 7.4, respectively. GSH: 10 mM. NIR irradiation:1064 nm at 1 W/cm^2^ for 5 min at regular intervals). *n* = 3 independent experiments.

Fourier transform infrared spectroscopy (FTIR) also confirmed the chemical bonds of Lipid 2-DG@Au/DOX-ss (Fig. 1F). The absorption peaks of Lipid 2-DG@Au/DOX-ss at 805 and 1220 cm^−1^ originated from the asymmetric stretching vibration of C-O-C of DOX. Other characteristic peaks at 1285 and 1412 cm^−1^ were attributed to the C-OH and C—C of DOX, the wavenumber at 1614 cm^−1^ was ascribed to the stretching vibration peak of bi‑carbonyl on the benzene ring of DOX (Qin et al., 2024). The stretching vibration of S—S peak was linked to the peaks at 539 cm^−1^. The characteristic peak at 1739 cm^−1^ was linked to the carbonyl stretching vibration of DSPE-PEG2000 (Alamri et al., 2024). The absorption peaks at 2918 cm^−1^ and 2854 cm^−1^ represented the alkyl of DSPE-PEG2000 (Ding and Kim, 2026; Li et al., 2024). The characteristic peak at 765 cm^−1^ originated from Au—S bond between Au NRs and Lipid DOX-ss (Cerra et al., 2026; Seino et al., 2024). The strengthen peaks of -OH, -CH-, -C=O- and -C=N- demonstrated the chain of 2-DG and the formation of pH-sensitive Schiff base group from exposed amino group and 2-DG. The encapsulation efficiency and DOX loading efficiency were calculated as 94.8% ± 4.37% and 26.70% ± 5.97%, respectively.

The photothermal conversion curves (Fig. 1G) showed no significant change over four near-infrared irradiations, indicating excellent photothermal stability of Lipid 2-DG@Au/DOX-ss. The photothermal conversion efficiency (ŋ) of Au NRs was measured to be 64.33% under 1064 nm laser irradiation (ŋ_808 nm_:46.52%), Lipid 2-DG@Au/DOX-ss (ŋ_1064 nm_:57.41%, ŋ_808 nm_:45.29%) was good enough to rival Au NRs. The promising photothermal conversion capability and stability of Lipid 2-DG@Au/DOX-ss makes it an excellent NIR-II photothermal nanomaterial.

Then the drug release process of Lipid 2-DG@Au/DOX-ss was investigated to assess the pH-GSH-NIR-II responsive release at 37 °C for 72 h (Fig. 1H). The cumulative release of DOX at pH 5.5 was about 61.66%, ascribing to the Au—S fracture by acid-responsive degradation (Lin et al., 2025). In contrast, the cumulative DOX release of Lipid 2-DG@Au/DOX-ss at pH 5.5 + NIR + GSH increased to 71.13% and 96.56% respectively. The DOX was almost entirely released from Lipid 2-DG@Au/DOX-ss via the acid, reduced, and photothermal stimulation, accelerating drug molecular movement and nanoparticle degradation, thus promoting drug release.

### 3.2 Target selectivity of Lipid 2-DG@Au/DOX-ss against MCF-7/ADR

The cell viability in L-02 cells and MCF-7/ADR cells were above 80% when the concentration of Au NRs and liposome exceeded 200 μg/mL at 24 and 48 h, respectively, indicating no appreciable cytotoxicity at the measured concentrations (Fig. 2A). Furthermore, the hemolysis rate of Lipid 2-DG@Au/DOX-ss was less than 5% even at a high concentration of 500 μg/mL (Fig. 2B), indicating negligible hemolytic effect on erythrocytes and a significant biocompatibility.

**Fig. 2:**
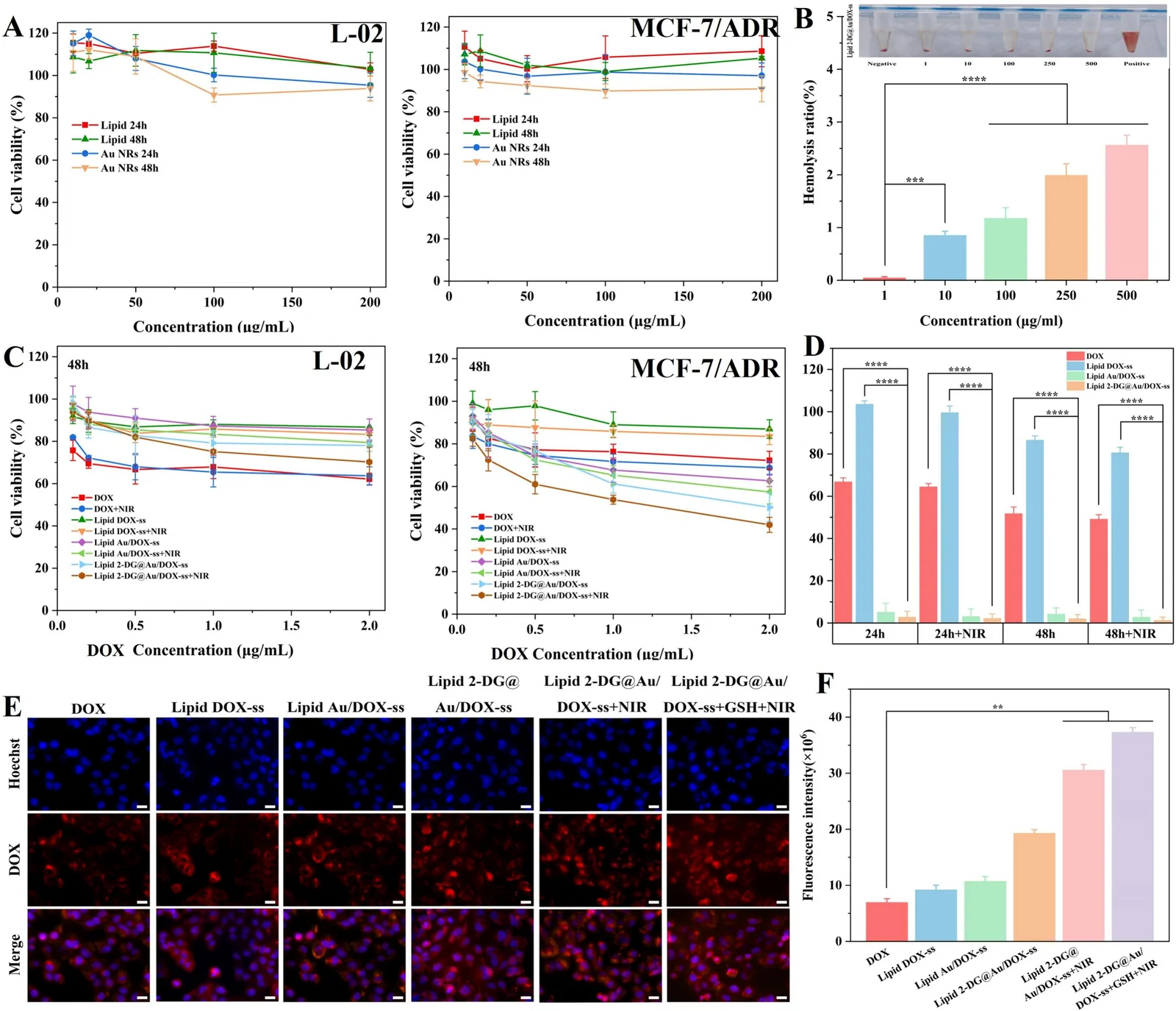
Detoxification of Lipid 2-DG@Au/DOX-ss on DOX-induced cytotoxicity. (A) In vitro cytotoxicity of various unloaded drug formulations against L-02, MCF-7/ADR cells at 24 and 48 h (*n* = 5). (B) Hemolysis determination and hemolysis percentage of Lipid 2-DG@Au/DOX-ss at different concentrations (*n* = 3). Negative control was sample that treated with saline, positive control was sample that treated with TritonX-100. (C) In vitro cytotoxicity of various drug-loaded formulations against L-02 and MCF-7/ADR cells at different DOX equivalent concentrations for 48 h (*n* = 5). (D) IC_50_ values in MCF-7/ADR cells (*n* = 5). (*E*-F) Fluorescence micrographs and quantitative analysis after endocytosis. Data are presented as mean ± standard deviation (SD, *n* = 3). Student's *t-*test, *p < 0.05, **p < 0.01, ***p < 0.001, ****p < 0.0001 indicate the statistically significant and extremely statistically significant, respectively.

The cell viabilities of L-02 cells treated with 2 μg/mL of DOX alone were 68.12% and 62.1% at 24 h and 48 h, respectively (Fig. 2C-D), indicating the significant toxicity of DOX to normal cells. Meanwhile, Lipid 2-DG@Au/DOX-ss showed better protection to cells at the same DOX equivalent concentration, indicating its excellent biocompatibility to mitigate the DOX cytotoxicity. Compared with DOX alone (68.91%), the cell viability of MCF-7/ADR cells with Lipid Au/DOX-ss and Lipid 2-DG@Au/DOX-ss (DOX equivalent concentration, 2 μg/mL) treatment were significantly decreased to 57.43% and 41.95% after 48 h incubation, respectively. The IC50 of DOX alone, Lipid DOX-ss, Lipid Au/DOX-ss, Lipid 2-DG@Au/DOX-ss and Lipid 2-DG@Au/DOX-ss under NIR laser stimulation after 48 h incubation were 51.67 μg/mL, 86.50 μg/mL, 4.13 μg/mL, 1.99 μg/mL and 1.17 μg/mL, respectively. These observations are consistent with the behavior of the densely packed liposomes at 37 °C, further validating the accelerated drug release triggered by the synergistic effects of Au—S bond-mediated pH-sensitivity, redox responsiveness, and NIR-II photothermal stimulation, alongside the DPPC phase transition above 41 °C.

As shown in Figs. 2E-F and S2A-B, red fluorescence began to emerge within the cytoplasm at 4 h. As the incubation time extended to 24 h, the fluorescence intensity in the perinuclear region progressively intensified, demonstrating the time-dependent endocytosis accumulation in MCF-7/ADR cells. Compared to the DOX alone group, the Lipid DOX-ss and Lipid Au/DOX-ss group exhibited therapeutic efficacy through the liposomal protection from premature drug leakage and the inherently limited EPR effect. Lipid 2-DG@Au/DOX-ss presented a markedly higher intracellular fluorescence intensity, due to the 2-DG-mediated glycolysis inhibition on effective depletion of ATP pools (Dai et al., 2026). Meanwhile, dual-stimulation with GSH and NIR-II laser resulted in 4.43-fold and 5.39-fold enhancement of fluorescence in Lipid 2-DG@Au/DOX-ss group over the DOX alone group after 4 h and 24 h, further confirmed the redox-triggered cleavage of disulfide linkages and photothermal-accelerated drug liberation. Coupled with 2-DG, GSH and NIR-II irradiation, the localized hyperthermia significantly transcends to increase membrane permeability and fluidity, thereby augmenting intracellular internalization, increasing drug influx and potentiation of bypassing pharmacological barriers of MCF-7/ADR cells (Muller et al., 2024; Wang et al., 2026).

### 3.3 2-DG dose-dependent metabolic crisis

As illustrated in Figs. 3A, C and S3A, C, the DOX alone group exhibited minimal ROS generation. However, Lipid 2-DG@Au/DOX-ss under GSH conditions showed a 24.01-fold increase in ROS fluorescence intensity relative to the control (Figs. 3C and S3C). This potent ROS burst suggests maximized oxidative stress for driving tumor apoptosis. Continuous intracellular ROS accumulation subsequently exacerbated mitochondrial impairment, intrinsically coupled with the dissipation of mitochondrial membrane potential. Correspondingly, the significant attenuation of green fluorescence in Lipid 2-DG@Au/DOX-ss under GSH and NIR-II stimulation (Figs. 3B, D and S3B, D) confirms rapid and severe mitochondrial dysfunction, marking a critical transition from metabolic stress to programmed cell death. This process is underpinned by the rapid disulfide bond cleavage and NIR-II-responsive drug liberation, guaranteeing targeted cargo delivery.

**Fig. 3:**
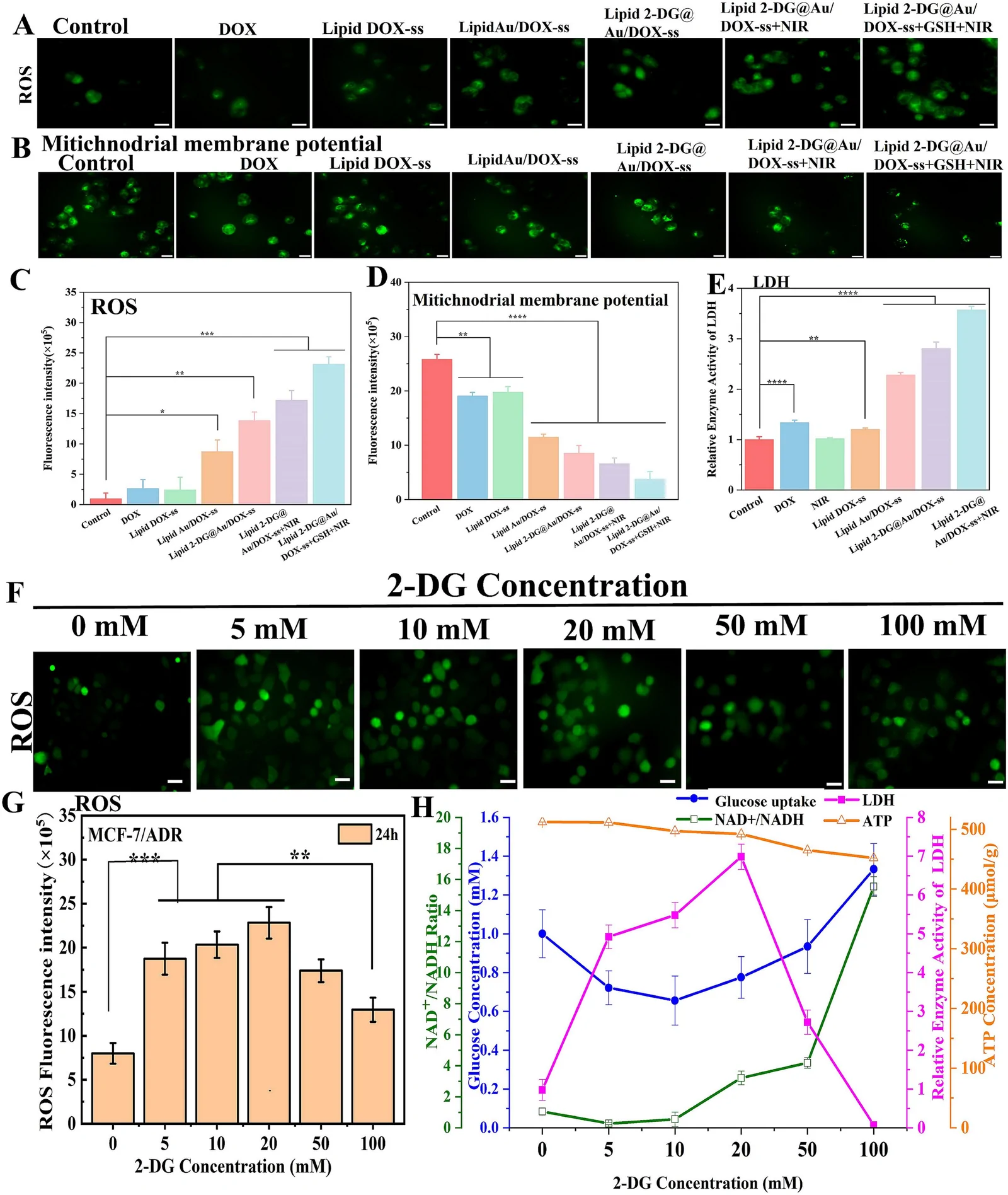
Nanoprodrug-induced metabolic crisis. (A) Intracellular ROS fluorescence of MCF-7/ADR cells after different treatment. *n* = 3. (B) Mitochondrial membrane potential via confocal microscopy. *n* = 3. (C-D) Quantitative fluorescence intensity of ROS and mitochondrial membrane potential. *n* = 3. (E) LDH related enzyme activity. *n* = 3 independent experiments. Control group was the untreated group that MCF-7/ADR cells were incubated with fresh medium. (F-G) Effects of 2-DG concentration of Lipid 2-DG@Au/DOX-ss on intracellular ROS fluorescence. (H) Dynamic alterations of metabolic indicators of glucose uptake, NAD^+^/NADH ratio, LDH and ATP levels. Scale bar:20 μm. Data are presented as mean ± standard deviation (SD, *n* = 3). Student's *t-*test, *p < 0.05, **p < 0.01, ***p < 0.001, ****p < 0.0001 indicate the statistically significant and extremely statistically significant, respectively.

Glycolysis inhibitor 2-DG has been reported to suppress nicotinamide (NAD^+^/NADH) phosphoribosyl transferase and attenuate pyruvate flux via the cytosolic enzyme reaction of lactate dehydrogenase (LDH). However, LDH leakage from cytoplasm into culture medium typically serves as a definitive indicator of compromised cell membrane integrity that precedes apoptosis or necrosis (Chen et al., 2024; Khaled et al., 2024). Notably, Lipid 2-DG@Au/DOX-ss with NIR-II irradiation exhibited a 3.57-fold increase in LDH leakage at 24 h (Figs. 3E and S2C), further confirming the extensive disruption of cell membrane integrity. Glycolysis inhibitor 2-DG has been reported to further suppress nicotinamide (NAD^+^/NADH) phosphoribosyl transferase and attenuate pyruvate flux via lactate dehydrogenase (LDH).

For this purpose, we examine the effect of 2-DG concentrations of Lipid 2-DG@Au/DOX-ss on ATP generation and NAD^+^/NADH (Fig. 3G-H). When the 2-DG concentration extended to 100 mM, the cell viability of MCF-7/ADR cells were significantly decreased from 72.45% to 49.67% after 48 h incubation of Lipid 2-DG@Au/DOX-ss (Fig. S4). Meanwhile the glucose uptake and NAD^+^/NADH ratio exhibited a transient decrease and long-term elevation. Conversely, the intracellular ROS and LDH accumulation followed a diametrically opposed trajectory. Compared with the PBS treated group, the intracellular ATP levels of Lipid 2-DG@Au/DOX-ss group declined persistently. The above non-monotonic alterations of glucose uptake, ATP, NAD^+^ and LDH indicate a profound disruption of metabolic homeostasis.

Thereby, these complex profiles are likely attributable by implementing a metabolic reprogramming strategy via 2-DG in MCF-7/ADR cells, triggering significant bioenergetic perturbations, characterized by non-monotonic alterations in glucose uptake, ATP, NAD^+^, and LDH levels.

### 3.4 In vivo anti-tumor efficacy and systematic dismantling of signaling pathway

As shown in Fig. 4A, the maximum temperature of control group (saline treatment) maintained at 33–35 °C, the tumor temperature of Lipid 2-DG@Au/DOX-ss (1064 nm 0.5 W/cm^2^, 5 min) rapidly rosed to over 50 °C, enabling ablation of tumor cells via thermotherapy. Fig. 4B-D showed that the tumor growth of DOX alone and Lipid DOX-ss groups exhibited marginal decline after 15 days treatments. These findings align with the in vitro cytotoxicity results, stemming from the thermo-responsive dynamics of DPPC-based liposomes and the intrinsic chemoresistance of MCF-7/ADR cells. Lipid 2-DG@Au/DOX-ss with NIR stimulation exhibited remarkable antitumor performance, the tumor volume and tumor weight were greatly reduced (on average about 226 mm^3^ and 0.28 g, NS group (saline): 1301 mm^3^ and 1.63 g).

**Fig. 4:**
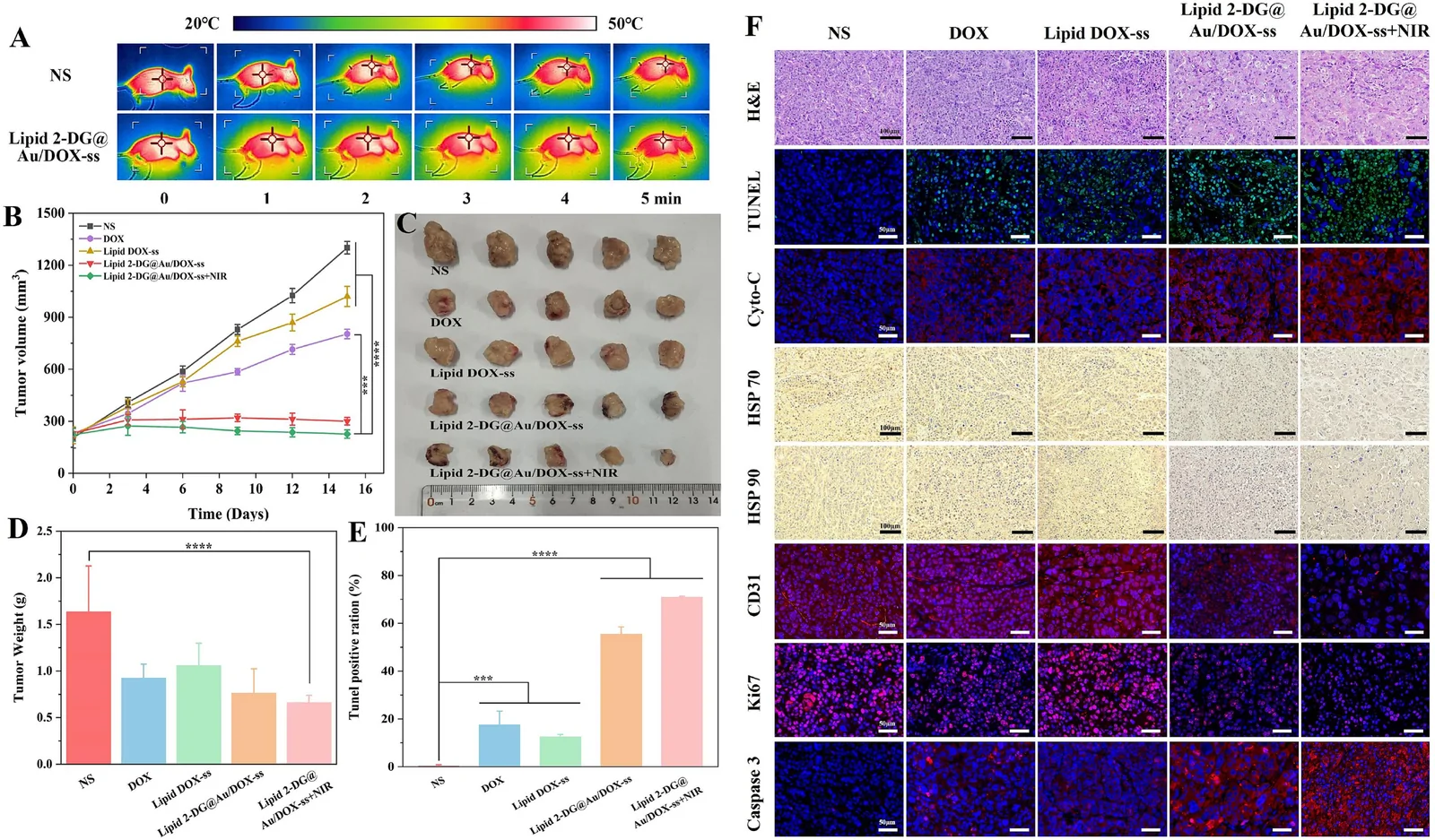
In vivo anti-tumor performance within 15 days. (A) In vivo photothermal images after Lipid 2-DG@Au/DOX-ss with 0.5 W/cm^2^, 1064 nm laser irradiation for 5 min. *n* = 5. (B) Tumor volume. *n* = 5. (C) Representative photographs of tumors extracted after the mice were sacrificed on day 15. *n* = 5. (D) Tumor weight. *n* = 5. (E) TUNEL-positive ratio. *n* = 3. (F) H&E, TUNEL, Cyto-C, HSP70, HSP90, CD31, Ki67 and Caspase 3 immunofluorescence staining of tumor slices (*n* = 6, scale bar: HE, HSP70 and HSP90: 100 μm. TUNEL, Cyto-C, CD31, Ki67 and Caspase3: 50 μm). NS group was the normal control group that mice were injected with saline. Data are presented as mean ± standard deviation (SD, *n* = 3). Student's *t-*test, *p < 0.05, **p < 0.01, ***p < 0.001, ****p < 0.0001 indicate the statistically significant and extremely statistically significant, respectively.

To further reveal the mechanism of antitumor effects, the H&E, TUNEL, Cyto-C, HSP70, HSP90, CD31, Ki67 and Caspase3 staining were performed on the tumor tissues after treatment (Fig. 4E-F). H&E and TUNEL images showed that the Lipid 2-DG@Au/DOX-ss under NIR laser stimulation had the most severe tumor tissue damage (tumor apoptosis about 70.85%; NS group: 0.22%), with tumor cell membranes rupture, organelles lysis and cell morphology alteration. Immunohistochemical results showed that the expression of HSP90 and HSP70 were dramatically decreased in the Lipid 2-DG@Au/DOX-ss under NIR laser stimulation, indicating the ATP inhibition-induced mitochondrial autophagy damage and HSPs depletion. Meanwhile, Lipid 2-DG@Au/DOX-ss under NIR laser stimulation showed the lowest Ki67 cell proliferation activity, indicating its ability to reduce tumor aggressiveness. Cyto-C staining results also showed the same trend, as the Lipid 2-DG@Au/DOX-ss under NIR laser stimulation indicated the most severe mitochondrial damage, leading to significant release of cytochrome C from mitochondrial and subsequent activation of caspase cascade. The autophagy regulatory of Caspase3 were consistent with the apoptosis of TUNEL assay that Lipid 2-DG@Au/DOX-ss under NIR laser stimulation exhibiting the highest apoptosis effect. Mitochondria play a significant role in regulating apoptosis or non-apoptotic cell death, and mitochondrial dysfunction can affect the release of cytochrome C from mitochondria, directly strikes at the biosynthetic heart of the tumor in apoptosis (Ding et al., 2026).

Additionally, CD31 expressions could be used to evaluate tumor angiogenesis and tumor growth speed. The significant inhibition of CD31 in Lipid 2-DG@Au/DOX-ss under NIR laser stimulation indicated the suppression of blood vessel development, which may further exacerbate aerobic respiratory pathway in the tumor site.

To further elucidate how this metabolic reprogramming translates into a global disruption of survival signaling in vivo, we performed comprehensive proteomic profiling on the harvested tumor tissues. The screening criteria for differentially expressed up or down-regulated proteins were |log2FC| ≥1, *p* value <0.05. Fig. 5A showed that there were 482 differentially expressed proteins in the Lipid 2-DG@Au/DOX-ss + NIR group compared with the control group, among which 243 proteins were up-regulated, and 239 proteins were down-regulated. Differentially expressed proteins were enriched and analyzed using Gene ontology (GO) and Kyoto encyclopedia of genes and genomes (KEGG) (Fig. 5B-F). The results of KEGG enrichment analysis were significantly enriched with many signaling pathways, including phagosome, FcγR-mediated phagocytosis, the NF-κB signaling pathway (Fig. 5B) and the TCA cycle, apoptosis, and phagosome signaling pathways (Fig. 5F). Cellular components (CC, Fig. 5C) were mainly enriched in plasma membrane protein complexes, plasma membrane, cytoplasmic vesicles, and intracellular vesicle. Molecular functions (MF, Fig. 5D) were mainly enriched in heparin binding, transmembrane signaling receptor, MHC class II protein complexes, oxidoreductases and NAD or NADP, consistent with a molecular switch of HSP70 to modulate autophagy pathways and antigen delivery for MHC class II presentation during nutrient stress (Skrabalak et al., 2025). Biological processes (BP, Fig. 5E) were mainly enriched in ROS metabolism, wound healing, macrophage cytokine, mitochondrial outer membrane permeabilization involved in programmed cell death and phagocytosis-related regulation. These metabolic alterations, coupled with an escalation in oxidative stress, ultimately trigger autophagy through the inhibition of the mTOR signaling pathway. GO and KEGG enrichment analyses demonstrated that differentially expressed proteins were significantly enriched for cellular components, molecular functions, and biological processes associated with protein efflux, angiogenesis, and autophagy.

**Fig. 5:**
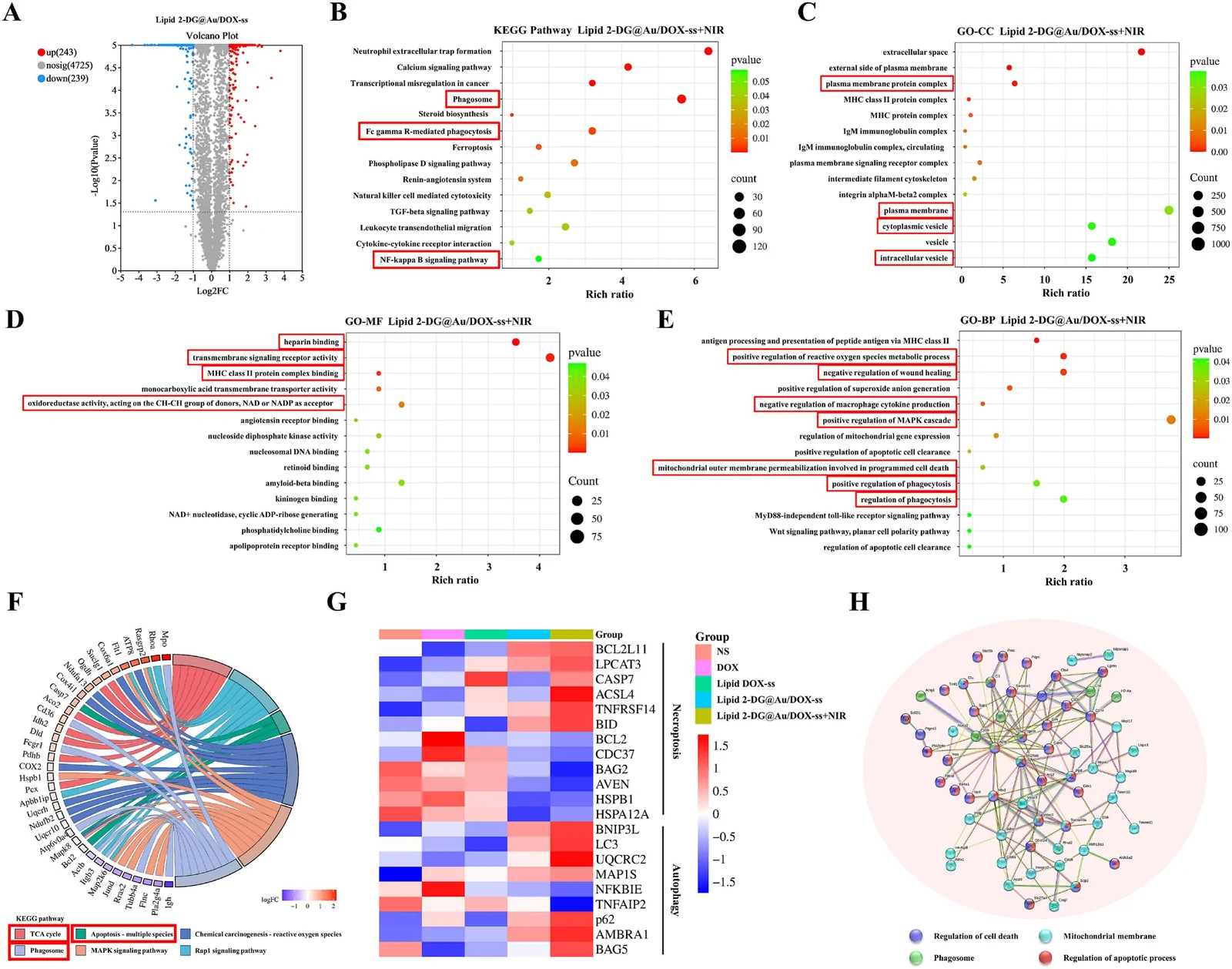
Proteomic profiling of tumor tissues. (A) Volcano plot of differential protein changes. (B) Enrichment analysis of differentially expressed proteins in the KEGG pathway. (C-E) Enrichment analysis of differentially expressed proteins in the GO pathway including CC(C), MF(D) and BP(E) processes. (F) KEGG-enriched chordal maps of differentially expressed proteins. (G) Heatmap of proteins associated with mitochondrial autophagy and cell death mode (apoptosis or necrotic apoptosis) in MCF-7/ADR-transplanted tumors after different treatments. Red-color represents up-regulation and blue-color indicates down-regulation. (H) PPI (protein-protein interaction) network of the identified differentially expressed proteins. (For interpretation of the references to color in this figure legend, the reader is referred to the web version of this article.)

Subsequently, key proteins associated with apoptosis, mitochondrial autophagy and mitochondrial membrane permeability transition pore were further analyzed (Fig. 5G-H), and several proteins associated with programmed cell death, heat stress, multidrug resistance, autophagy as well as mitochondrial autophagy. Functional enrichment further uncovered differentially potential systemic perturbations in immune, ferroptosis and calcium overload signaling via ACSL4/LPCAT3 pathway(Lu et al., 2025; Zhuang et al., 2025). CASP7, BCL2, HSPB1 HSPA12A, CDC37 were significantly down-regulated, LC3, p62 and AMBRA1 (a tumor suppressor protein, as part of autophagy and cell-cycle regulatory network) were significantly up-regulated. HSPA12A, an atypic member of HSP70 family, is related to c-Myc lactylation-mediated nuclear localization via glycolysis-derived lactate generation in a Hif1 alpha-dependent manner to cell proliferation. CDC37 (Cell division cycle 37) is HSP90 kinase-specific co-chaperone to disrupt abnormal client proteins and cell growth. These data demonstrate that the anti-tumor effect of Lipid 2-DG@Au/DOX-ss stems from the concurrent inhibition of HSP A12A(70)/90/B1(27)-mediated protein folding and the disruption of mitochondrial autophagic clearance.

### 3.5 Nanoprodrug-mediated S-phase arrest and mitophagy-associated apoptosis

As shown in Fig. 6A, Lipid 2-DG@Au/DOX-ss gradually approached and tended to enter the cells after 30 min, indicating the initiation of endocytosis. After 3 h incubation, Lipid 2-DG@Au/DOX-ss could enter the lysosomes. Interestingly, Lipid 2-DG@Au/DOX-ss passed into the mitochondria after 24 h, the mitochondria were slightly swollen, the mitochondrial cristae disappeared, and even vacuolization occurred, demonstrated the damage of mitochondrial membrane and the presence of prominent autophagosomes. Moreover, Lipid 2-DG@Au/DOX-ss was gradually excreted and observed in both intracellular and extracellular cells after 48 h, at the same time, MCF-7/ADR cells exhibited apoptotic characteristic features, the intact plasma membrane displays blistering morphology. The above results revealed the convergence of mitophagy flux impairment and mitochondrial apoptosis.

**Fig. 6:**
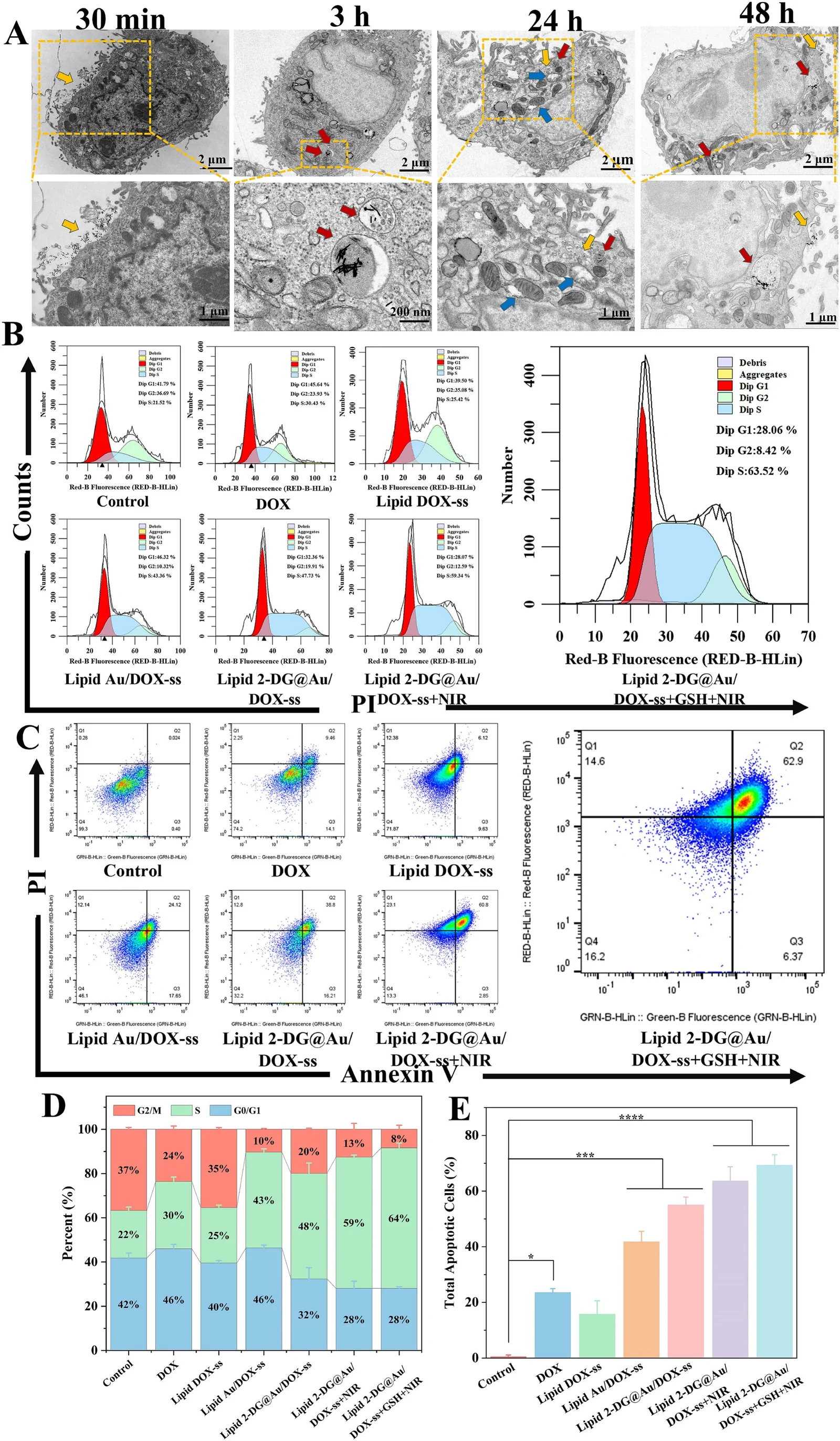
Nanoprodrug-mediated S-phase arrest and mitophagy-associated apoptosis (A) Bio-TEM images of MCF-7/ADR cells after different treatments. Yellow arrow: Lipid 2-DG@Au/DOX-ss endocytosis; Red arrow: Autophagosomes and autolysosomes; Blue arrow: mitochondrial dysfunction. (B) Cell cycle arrest of MCF-7/ADR cells. (C) Flow cytometry analysis of cell apoptosis after therapy. (D) Cell cycle arrest analysis. (E) Cell apoptosis analysis. Control group was the untreated group that MCF-7/ADR cells only incubated with fresh medium. GSH: 10 mM. NIR irradiation:1064 nm at 1 W/cm^2^ for 5 min. Data are presented as mean ± standard deviation (SD, *n* = 3). Student's *t-*test, *p < 0.05, **p < 0.01, ***p < 0.001, ****p < 0.0001 indicate the statistically significant and extremely statistically significant, respectively. (For interpretation of the references to color in this figure legend, the reader is referred to the web version of this article.)

Since DNA replication is a highly energy-consuming process, we further investigated whether the induced bioenergetic crisis and ROS burst affected cell cycle arrest. Flow cytometric analysis (Figs. 6B and S5A, C) revealed a most G2/M and G0/G1-phase arrest in the control group (the untreated group that MCF-7/ADR cells only incubated with fresh medium), a slight increase in S-phase arrest (reaching 30%) after DOX alone treatment, exhibiting drug resistance of MCF-7/ADR cells to DOX. However, Lipid 2-DG@Au/DOX-ss under GSH and NIR-II stimulation forced cells into robust S-phase arrest ((reaching 64%), potentially intensifying replication-associated DNA lesions.

Apoptosis rates of DOX alone were 8.83% and 23.56% at 12 h and 24 h, respectively (Figs. 6C and S5B, D). In contrast, the apoptosis rates of Lipid DOX-ss was slightly decreased, while Lipid 2-DG@Au/DOX-ss synergistically promoted a mitophagy-associated apoptosis (reaching 55.01% at 24 h), identifying the 1,2-dipalmitoyl-SN-glycerol-3-phosphoacylcholine-mediated thermosensitive drug release via limited fluidity and permeability of phospholipid membrane molecules below 41 °C (Hosseinikhah et al., 2025; Mousavi et al., 2026). The NIR and GSH dual-stimulation facilitates Lipid 2-DG@Au/DOX-ss a high apoptosis rate at 69.27%, revealing the bioenergetic crisis triggers irreversible commitment to cell death via S-phase arrest and DNA replication impairment.

### 3.6 Nanoprodrug-mediated HSPs-P-gp collapse and mitophagy flux impairment with ferroptosis

Mild hyperthermia activates the anti-apoptotic and self-protective programs (especially the heat shock response) to counteract thermal damage, thus significantly limiting the photothermal efficacy (Ding et al., 2025a). As shown in Fig. 7A-B, the P-glycoprotein (P-gp) expression of MCF-7/ADR cells after Lipid 2-DG@Au/DOX-ss and NIR-II laser stimulation was 89% lower than that of the control group (untreated).

**Fig. 7:**
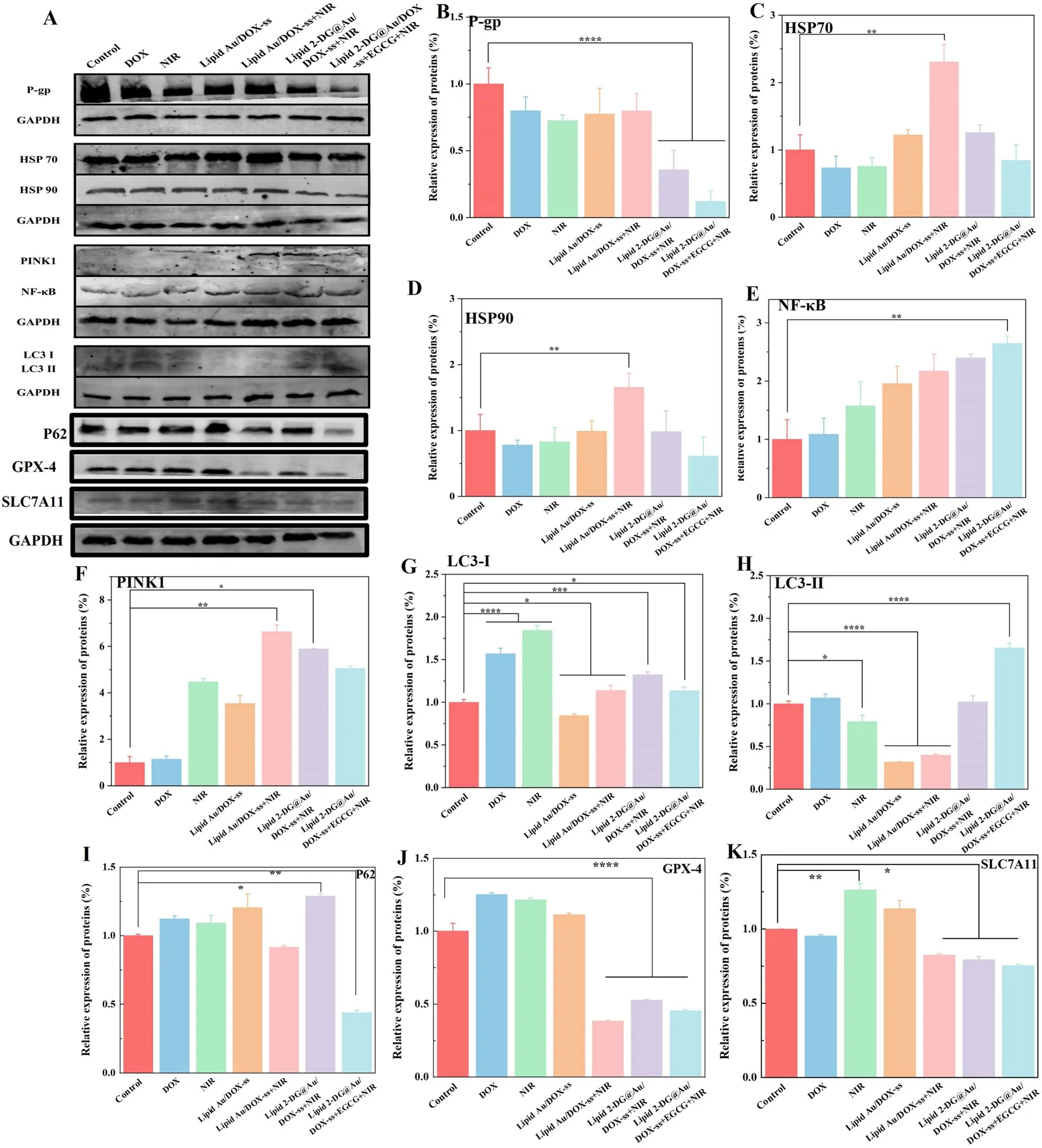
Molecular validation of the HSPs-P-gp axis collapse and mitophagy flux impairment in MCF-7/ADR cells via Western blot analysis. (A) Key proteins expression for HSPs, P-gp, mitophagy and ferroptosis regulation. (B-K) Quantitative expressions of P-gp, HSP70, HSP90, NF-κB, PINK1, LC3-I, LC3-II, p62, GPX-4 and SLC7A11. (EGCG: 50 μM GSH: 10 mM. NIR irradiation:1064 nm at 1 W/cm^2^ for 5 min). Control group was the untreated group that MCF-7/ADR cells were incubated with fresh medium. Data are presented as mean ± standard deviation (SD, *n* = 3). Student's *t-*test, *p < 0.05, **p < 0.01, ***p < 0.001, ****p < 0.0001 indicate the statistically significant and extremely statistically significant, respectively.

As shown in Fig. 7A-D, the expressions of HSP70 and HSP90 of Lipid Au/DOX-ss with NIR-II irradiation drastically increased 2.32 and 1.75-fold than the control group, indicating photothermal-induced protein-chaperone recruitment. While the expressions of HSP70 and HSP90 of Lipid 2-DG@Au/DOX-ss were 46% and 44% lower than Lipid Au/DOX-ss after NIR-II irradiation, where maintained at near-baseline values, indicating the 2-DG-induced bioenergetic crisis to threshold the halted protein synthesis and paralyze the tumor's adaptive thermotolerance^.^(Hong et al., 2026; Liu et al., 2025). Notably, the inclusion of HSP90 inhibitor epigallocatechin gallate (EGCG). resulted in a marginal reduction in HSP70 and HSP90 levels. These results highlight the superior bioenergetic deprivation of 2-DG, outperforming concurrent inhibition of HSP 70/90 protein folding.

Furthermore, the levels of NF-κB, PINK1, LC3-II and p62 of Lipid 2-DG@Au/DOX-ss group with NIR-II irradiation were 1.61-fold, 5.05-fold, 0.65-fold and 0.29-fold higher than the control group (untreated) (Fig. 7E-I), respectively. The elevated LC3-II and p62 together with the accumulation of PINK1 indicates the activation of mitophagy and blockade of the downstream of the autophagic flux as a compensatory response to the ATP depletion and HSP90 downregulation induced by our nano-prodrug. In addition, compared with the control group (untreated), the ferroptosis markers such as GPX4 and SLC7A11 showed significant reductions in the Lipid 2-DG@Au/DOX-ss group with NIR-II irradiation (with 48.33% and 21.67% reduction, respectively) (Fig. 7J-K).

Our findings demonstrate that Lipid 2-DG@Au/DOX-ss photothermal nano-prodrug-mediated bioenergetic crisis on suppressing glycolysis, ATP levels and glucose uptake. This metabolic stress leads to the collapsing of the proteostatic machinery, characterized by the significant downregulation of HSP70/HSP90. Simultaneously, the ATP depletion inhibits the efflux pump P-gp, thereby sensitizing resistant cells. On a molecular signaling level, this stress is transduced via the NF-κB pathway and initiates PINK1-dependent mitophagy, as reflected by the robust conversion of LC3-I to LC3-II. And the ferroptosis were further promoted to induce cancer cell apoptosis. Proteomics and Western blot analysis confirmed the synergistic alteration of NF-κB signaling, a massive ROS burst, mitophagy flux impairment, and ferroptosis regulation.

### 3.7 Histopathological evaluation and in vivo therapeutic mechanism of nanoprodrug

Fig. 8A showed that the mice weight in the DOX alone group gradually decreased after five administrations, indicating the obvious systemic toxicity of DOX. There were no abnormal fluctuations in the body weights of mice throughout the observation period, indicating the negligible adverse effects of drug-loaded nanoparticles.

**Fig. 8:**
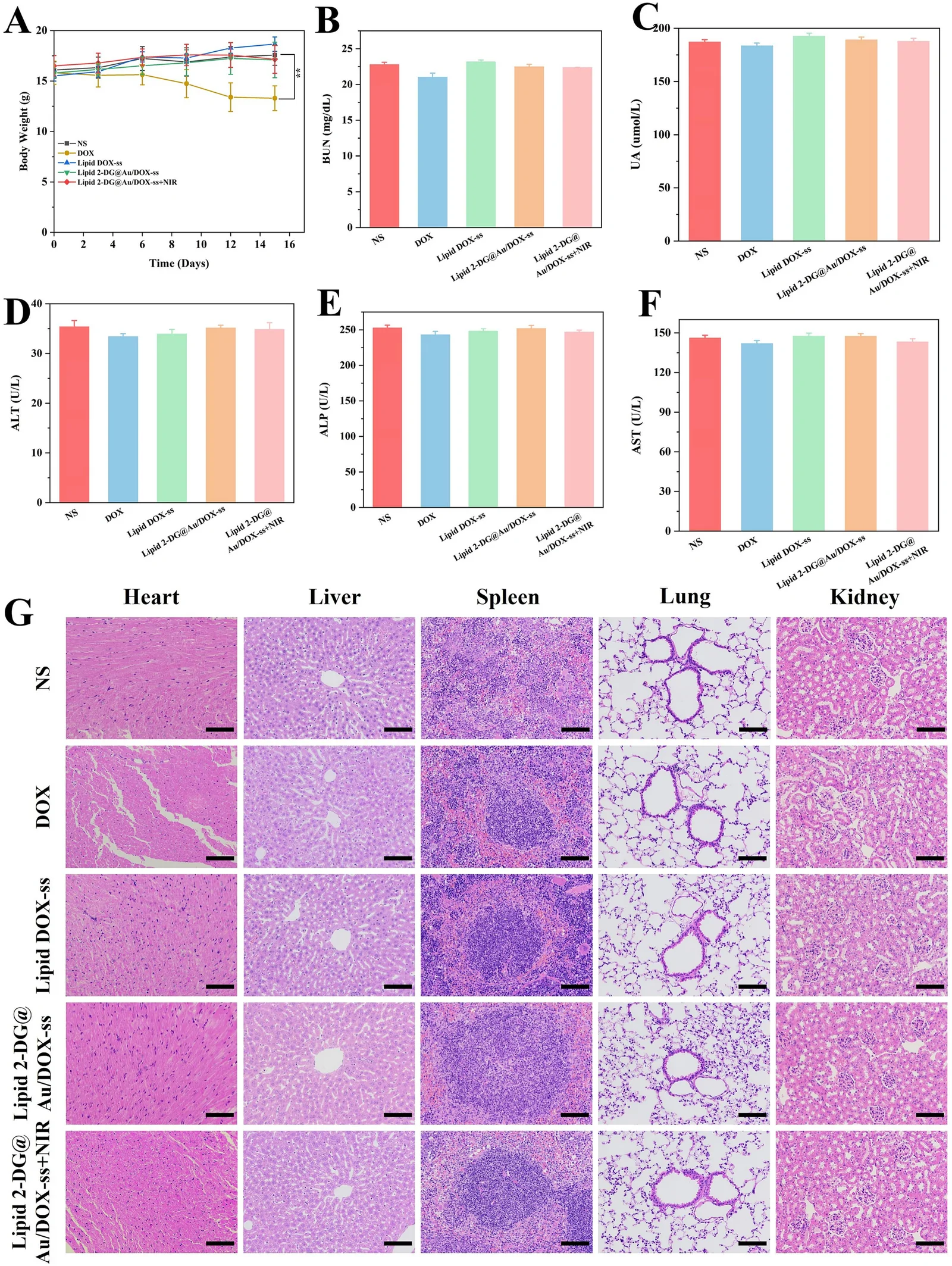
Histopathological evaluation of nanoprodrug. (A) Body weight curves of mice under different treatment methods within 15 days. (B-C) Serum biochemistry indices that reflect renal functions. (D-F) Serum biochemistry indices that reflect hepatic functions. (G) H&E staining of the heart, livers, spleen, lung, and kidneys of mice (scale bar: 100 μm). NS group was the normal control group that the mice were only injected with saline solution. Data are presented as mean ± standard deviation (SD, *n* = 3). Student's *t-*test, *p < 0.05, **p < 0.01, ***p < 0.001, ****p < 0.0001 indicate the statistically significant and extremely statistically significant, respectively.

Hepatic and renal functions including alanine aminotransferase (ALT), aspartate aminotransferase (AST), alkaline phosphatase (ALP), blood urea nitrogen (BUN) and uric acid (UA) were also conducted to further assess any potential toxic effects (Fig. 8B-F). There were no significant differences in the serum biochemistry indices that reflect hepatic and renal functions, indicating the favorable biosafety of drug-loaded nanoparticles after 15 days treatment.

Furthermore, these results were also consistent with the histological analysis of H&E staining of vital organs (Fig. 8G). The DOX alone group exhibited a certain degree of cardiotoxicity, liver necrosis and kidney damage. HE tissues analysis is more sensitive in detecting early pathological changes or mild damage stages in major internal organs, while the serum biochemistry indicators usually only increase significantly after a certain degree of damage has occurred. The above results indicate that Lipid 2-DG@Au/DOX-ss at this dosage and administration frequency do not cause severe toxicity that threatens the survival of tumor-bearing mice.

Collectively, 2-DG-mediated metabolic exhaustion acts as a primary driver of Lipid 2-DG@Au/DOX-ss, not only compromises the ATP-dependent HSP-P-gp defense but also halts the S-phase arrest, mitophagy flux impairment and apoptosis via NF-κB/PINK1/LC3 pathway, ensuring eradication of MCF-7/ADR cells.

## 4 Conclusions

In summary, a pH-GSH-NIR-II nanoprodrug Lipid 2-DG@Au/DOX-ss was developed to overcome the intrinsic thermotolerance in MCF-7/ADR tumor. Coupled with 2-DG dose-dependent metabolic crisis metabolic perturbations and ROS cascade, the compensatory feedback loops of HSPs-P-gp were abrogated, specifically suppressed the HSP70 family member HSPA12A (as identified by proteomics) and HSP90. Meanwhile, mitophagy flux impairment was initiated to culminate a robust S-phase arrest and irreversible apoptosis via NF-κB/PINK1/LC3-II pathway. Proteomic analysis unveils the involvement of immune modulation, ferroptosis, and calcium homeostasis. Collectively, this redox-metabolic cascade NIR-II prodrug provides a potent and precise strategy for eradicating intrinsic thermotolerance of MDR malignancies via compensatory HSPs/P-gp with mitophagy flux impairment.

## CRediT authorship contribution statement

**Lan Cui:** Writing – review & editing, Supervision, Investigation, Funding acquisition. **Weishuang Lou:** Writing – original draft, Software, Methodology, Formal analysis. **Wencan Zhou:** Writing – original draft, Validation. **Mengyao Sun:** Visualization, Validation, Formal analysis, Data curation. **Zhongchen Jiao:** Validation, Formal analysis, Data curation. **Mengdi Li:** Formal analysis. **Tong Wang:** Formal analysis. **Xinyu Liang:** Formal analysis. **Xinmeng Jing:** Formal analysis. **Shuoye Yang:** Methodology, Data curation. **Pengshuai Zhang:** Writing – review & editing, Supervision, Project administration. **Lingbo Qu:** Supervision. **Peng Li:** Supervision, Methodology.

## Ethics declaration

### Animal subject

Female BALB/C nude mice (4 weeks old, about 16 ± 2 g, No.110011230106633484) were purchased from Beijing Vital River Laboratory Animal Technology Co., Ltd. (Beijing, China). Animal experiments were approved and performed following the animal protocol (license number, ZZU-LAC20241014[02]) in accordance with the guidelines of the Animal Center Committee of the National Drug Safety Evaluation Research Center of Zhengzhou University (Zhengzhou, China).

## Declaration of competing interest

The authors declare that they have no competing financial interests in this paper.

## Data Availability

Data will be made available on request.

## References

[bb0005] Abbott K.L., Subudhi S., Ferreira R., Gültekin Y., Steinbuch S.C., Bin Munim M., Ramesh D.L., Honeder S.E., Kumar A.S., Wu M., Hansen J.A., Shevzov-Zebrun A., Rashan E.H., Eghbalian K.M., Sivanand S., Barbeau A.M., Riedmayr L.M., Duquette M., Ali A., Henning N., Trojan S.E., Waite M., Kunchok T., Nakano M.A., Gourgue F., Ferraro G.B., Do B.T., Spanoudaki V., Sánchez-Rivera F.J., Jin X., Church G.M., Jain R.K., Vander Heiden M.G. (2026). Nutrient requirements of organ-specific metastasis in breast cancer. Nature.

[bb0010] Alamri O.A., Qusti S., Balgoon M., Ageeli A.A., Al-Marhaby F.A., Alosaimi A.M., Jowhari M.A., Saeed A. (2024). The role of MoS2 QDs coated with DSPE-PEG-TPP in the protection of protein secondary structure of the brain tissues in an Alzheimer’s disease model. Int. J. Biol. Macromol..

[bb0015] Cerra S., Ranaldi M., Amatori S., Fioravanti R., Milanese F., Salamone T.A., Pennacchi B., Mercurio M., Battocchio C., Marsotto M., Nappini S., Capocefalo A., Fasolato C., Scaramuzzo F.A., Davydok A., Grigorian S., Thomas O., Texier M., Fratoddi I. (2026). Tuning the optoelectronic features of poly(3-hexylthiophene-2,5-diyl) with oligoplatinaynes-functionalized gold nanoparticles. ACS Appl. Nano Mater..

[bb0020] Chen Y.P., Wu J.H., Zhai L.H., Zhang T.T., Yin H., Gao H.Y., Zhao F., Wang Z., Yang X.N., Jin M.P., Huang B.S., Ding X., Li R., Yang J., He Y.M., Wang Q.W., Wang W.B., Kloeber J.A., Li Y.X., Hao B.B., Zhang Y.Y., Wang J.D., Tan M.J., Li K., Wang P., Lou Z.K., Yuan J. (2024). Metabolic regulation of homologous recombination repair by MRE11 lactylation. Cell.

[bb0025] Cui L., Xu Q.Q., Lou W.S., Wang Y.L., Xi X.L., Chen Y.L., Sun M.Y., Wang Z.H., Zhang P.S., Yang S.Y., Zhang L., Qu L.B. (2024). Chitosan oligosaccharide-functionalized nano-prodrug for cascade chemotherapy through oxidative stress amplification. Int. J. Biol. Macromol..

[bb0030] Dai J.N., He Y.C., Sun M.M., Cao Y., Wang W., Feng B.H., Xie S.C., Hua J., Wang J., Ma P.A., Fang L.P. (2026). Targeting energy-metabolism-imbalance nanoagonist for high-resolution NIR-II imaging and potentiated photoimmunotherapy. Adv. Funct. Mater..

[bb0035] Ding Q., Kim J.S. (2026). Molecularly intelligent photosensitizers: pioneering a new era in photodynamic therapy. Sci. China Chem..

[bb0040] Ding Q.H., Chen S.Y., Hua S., Yoo J., Yoon C., Li Z.Q., Zhao E.G., Kim J.S., Gu M.J. (2025). Photoactivated nanovaccines. Chem. Soc. Rev..

[bb0045] Ding Q.H., Liu J.Q., Wang Y., Kim J., Huang Z.H., Lee Y.B., Zhou H., Zhang P.F., Sessler J.L., Kim J.S. (2025). Dual- laser “808 and 1,064 nm” strategy that circumvents the Achilles’ heel of photothermal therapy. Proc. Natl. Acad. Sci. USA.

[bb0050] Ding Q.H., Qi M.L., Li W., Li M.Q., Xu J.L., Kim Y., Kim G., Dong B., Wang L., Kim J.S. (2025). Precision phototherapy enabled by decoding complex microenvironments. Acc. Chem. Res..

[bb0055] Ding Q.H., Li M.Q., Mei L., Hua S.W., Li Z., Mengji R., Chen R.Z., Liu H.W., Kim G., Jia M.X., Wang B., Yin C., Fan Q.L., Zhou H., Hu W.B., Cui J.W., Kim J.S. (2026). Immune-evasive NIR-II nanoplatforms for repeatable photodynamic therapy. J. Am. Chem. Soc..

[bb0060] Harter P., Marmé F., Redondo A., Reuss A., Lindemann K., Kurzeder C., Van Nieuwenhuysen E., Marth C., Pietzner K., Ray-Coquard I., Garcia-Duran C., Jonuskiene G., Heitz F., Béllier C., Pérez-Fidalgo J.A., El-Balat A., Selle F., Romero I., Wimberger P., Follana P., Pardo B., de Gregorio N., Joly F., Gaba L., Burges A., Fabbro M., Hasenburg A., Fehm T., Schmalfeldt B., Pautier P. (2026). Atezolizumab with bevacizumab and nonplatinum chemotherapy for recurrent ovarian cancer: final results from the placebo-controlled AGO-OVAR 2.29/ENGOT-ov34 phase III trial. J. Clin. Oncol..

[bb0065] Hong Z.W., Liu X.L., A R., Chen Z.Z., Wu Q.J., Fu J.X., Guan X., Wang C., Wang X.H., Jiang S., Li B.L., Wang Z.B., Zhang L., Meng X.W., Cui Y.L., Ma T.C. (2026). Disrupting fumarylacetoacetate hydrolase by stratified nanoplatforms orchestrates metabolic-immune reprogramming and prevents post-ablation HCC relapse. Nat. Commun..

[bb0070] Hosseinikhah S.M., Farhoudi L., Mirzavi F., Vahdat-Lasemi F., Arabi L., Gheybi F., Sazgarnia A., Alavizadeh S.H., Jaafari M.R. (2025). Ultrasound-assisted efficient targeting of doxorubicin to the tumor microenvironment by lyso-thermosensitive liposomes of varying phase transition temperatures. Eur. J. Pharm. Sci..

[bb0075] Khaled A.M., Othman M.S., Obeidat S.T., Aleid G.M., Aboelnaga S.M., Fehaid A., Hathout H.M.R., Bakkar A.A., Moneim A.E.A., El-Garawani I.M., Morsi D.S. (2024). Green-synthesized silver and selenium nanoparticles using berberine: a comparative assessment of in vitro anticancer potential on human hepatocellular carcinoma cell line (HepG2). Cells.

[bb0080] Li Y., Liao J., Xiong L.Y., Xiao Z.C., Ye F., Wang Y., Chen T., Huang L.Z., Chen M., Chen Z.S., Wang T.F., Zhang C., Lu Y. (2024). Stepwise targeted strategies for improving neurological function by inhibiting oxidative stress levels and inflammation following ischemic stroke. J. Control. Release.

[bb0085] Li J.G., Zhang Y.H., Shao C.Y., Bai Y.N., Wang P.X., Ren T.Y. (2025). Utilization of DOX-Fe complex and RSL3 co-loaded liposomes in ferroptosis-enhanced treatment of triple-negative breast cancer. Drug Deliv..

[bb0090] Lin J.Q., Huang X.M., Wang X.Z., Yu R.L., Liang J., Song R., Chen W.B., Shen G.L. (2025). RGD-modified gold nanoparticles loaded with SAHA remodel the hypoxic inflammatory microenvironment via inhibiting HIF-1α-VEGF signaling to enhance radiosensitivity in NSCLC. Int. J. Nanomedicine.

[bb0095] Liu Y.P., Yang Z., Huang L., Zeng W., Zhou Q. (2024). Anti-interference detection of mixed NOX via In2O3-based sensor array combining with neural network model at room temperature. J. Hazard. Mater..

[bb0100] Liu C.M., Cheng S.H., Zhou X., Li L., Wang C.G., Zhang L.Y. (2025). Mitochondrial dynamics and energy metabolism interference therapy for promoting photothermal sensitization. J. Colloid Interface Sci..

[bb0105] Liu Y.C., Wang P.H., Xia X.L., Gao W.H., Wang Y., Huang W., Yan D.Y. (2026). A smart P-gp inhibitor-drug conjugate nanomedicine overcomes administration challenges and multidrug resistance in breast cancer therapy. Biomater. Sci..

[bb0110] Lu Y.S., Zu Z.Y., Zhong N., Li Y., Sha X., Lu S.Y., Zhang J., Sun H.Y., Luo X., Xu Z.Q., Wu F.Y., Tang Y.X., Wang S.J. (2025). Spatiotemporal blockade of autophagy and macropinocytosis in pancreatic cancer via lysosomal-activated nanoreactors. Adv. Funct. Mater..

[bb0115] Mayaka R.K., Alocilja E.C. (2025). Genomic nano-biosensor for rapid detection of the carbapenem-resistant gene blaNDM-1 in carbapenemase-producing bacteria. Nanoscale Adv..

[bb0120] Mousavi S.M., Zhang Y., Pandzic E., Inglis D.W., Lord M.S., Taylor R.A., Li M. (2026). Uniform microscale thermoresponsive liposomes for controlled release above body temperature. Small.

[bb0125] Muller C., Lacroix-Malgras V., Kluza J., Laine W., Güler Y., Bost F., Boisbrun M., Mazerbourg S., Flament S. (2024). The troglitazone derivative EP13 disrupts energy metabolism through respiratory chain complex I inhibition in breast cancer cells and potentiates the antiproliferative effect of glycolysis inhibitntriors. Cancer Cell Int..

[bb0130] Qin S.L., Wen Z.W., Huang H.J., Wu W. (2024). Use of novel taurine-chitosan mediated liposomes for enhancing the oral absorption of doxorubicin via the TAUT transporter. Carbohydr. Polym..

[bb0135] Rembialkowska N., Rossowska J., Novickij V., Lapinska Z., Saczko J., Kulbacka J. (2026). Time-dependent stress responses determine bleomycin electrochemotherapy efficacy in drug-resistant breast cancer cells. Bioelectrochemistry.

[bb0140] Seino S., Ikehata H., Tanabe M., Umeda T., Tomiyama T., Tanaka A., Furubayashi T., Sakane T., Kiwa T., Washino M., Nomura K., Tonooka S., Izawa A., Okumura Y., Nakagawa T. (2024). Investigating the efficacy of nasal administration for delivering magnetic nanoparticles into the brain for magnetic particle imaging. J. Control. Release.

[bb0145] Shafeek F., El-Kashef D.H., Abu-Elsaad N., Ibrahim T. (2023). Epigallocatechin-3-gallate in combination with corticosteroids mitigates heat stress-induced acute kidney injury through modulating heat shock protein 70 and toll-like receptor 4-dependent pathways. Phytother. Res..

[bb0150] Skrabalak I., Rajtak A., Malachowska B., Skrzypczak N., Skalina K.A., Guha C., Kotarski J., Okla K. (2025). Therapy resistance: modulating evolutionarily conserved heat shock protein machinery in cancer. Cancer Lett..

[bb0155] Song S.L., Zhao Y., Kang M.M., Zhang F., Wu Q., Niu N., Yang H., Wen H.F., Fu S., Li X., Zhang Z.J., Tang B.Z., Wang D. (2024). An NIR-II excitable AIE small molecule with multimodal phototheranostic features for orthotopic breast cancer treatment. Adv. Mater..

[bb0160] Tang L.X., Wang S., Hu J., Meng L.L., Zhang J., Chang Y., Ma X.M., Guo Y.M. (2025). Rational design of Au-Bi bimetallic nanozyme for NIR-II laser mediated multifunctional combined tumor therapy. Colloids Surf. B: Biointerfaces.

[bb0165] Wang R., Cao H.C., Yang Q., Wei S., Liu T., Shi H. (2024). EGCG-vanadium nanomedicine with neutral pH Fenton reaction activity inhibits heat shock proteins for enhanced photothermal/ chemodynamic therapy. Int. J. Biol. Macromol..

[bb0170] Wang K.L., Ni C., Huang J.H., Huang Y.F., Cao X.Y., Ma J., Shi X.Y., Guo R. (2026). Integrated cascade catalysis of AuPtCu nanozymes and glycolysis inhibition for synergistic breast cancer therapy via metabolism regulation. Biomater. Sci..

[bb0180] Xu Y.X., Dang H.P., Teng C.C., Yin D.L., Yan L.F. (2024). ATP inhibition for starvation/mild photothermal/photodynamic synergy therapy using polypeptide nanoparticles conjugating 2-deoxy-D-glucose and dye under NIR phototheranostic strategy. Adv. Healthc. Mater..

[bb0185] Zhang W., Hu R.W., Yang R., De Rosa I.M., Yang J.M., Shen X.D., Kodambaka S., Xin W.B. (2026). Surfactant-free synthesis of mixed-phase (cubic plus hexagonal) gold and gold-graphene core-shell nanowires. New Carbon Mater..

[bb0190] Zhang F.F., Shang L., Zhang Z.L., Cheng Y.H., Liu H., Du X.W. (2026). Controllable synthesis of metal nanoparticles under non-equilibrium conditions via an extended LaMer mechanism. Small Methods.

[bb0195] Zhou H.L., Gao D., Cheng G.D., Wang N.N., Zhang R., Qiu L., Yuan H.B., Wang C.F., Gao H., Qu X.W., Xing C.F. (2025). Biofilm ablation on titanium alloy surface by photothermal and chemotherapeutic synergistic therapy. J. Mater. Sci. Technol..

[bb0200] Zhuang J.B., Song S.Y., Yang L.J., Pan Q., He Y., Huo L.T., Li N., Zhao N. (2025). NIR-II type I photosensitizer for efficient cancer therapy through synergistic ferroptosis/pyroptosis induction and mitophagy inhibition. Adv. Healthc. Mater..

[bb0205] Zou X., Zhang Y.H., Liu K.L., Zhang L.Y., Li J.L., Zhang Y., Zhang X.R., Yu L., Qu Z.Y. (2026). Chelidonine overcomes P-gp-mediated adriamycin resistance in MCF-7/ADR cells by inhibiting PDGFRβ/PI3K/Akt pathway. Chin. Herb. Med..

