# Supplementary material for: Redox-metabolic cascade NIR-II prodrug for synchronizing compensatory HSPs/P-gp feedback with mitophagy flux impairment against intrinsic thermotolerance
Source: Int J Pharm X. 2026 Sep 26;12:100671. doi: 10.1016/j.ijpx.2026.100671 (PMC13659683; doi:10.1016/j.ijpx.2026.100671)
Supplement: Supplementary file 1 [file mmc1.docx]

**Redox-metabolic cascade NIR-II prodrug for synchronizing compensatory HSPs/P-gp feedback with mitophagy flux impairment against intrinsic thermotolerance**

Lan Cui^a,b,c,^*, Weishuang Lou^a^, Wencan Zhou^a^, Mengyao Sun^a^, Zhongchen Jiao^a^, Mengdi Li^a^, Tong Wang^a^, Xinyu Liang^a^, Xinmeng Jing^a^, Shuoye Yang^a,c^, Pengshuai Zhang^a^, Lingbo Qu^d^, Peng Li^c,^*

*^a^ College of Biological Engineering,* *Henan University of Technology, Zhengzhou 450001, PR China.*

*^b^ School of Chemical Engineering, University of Birmingham, Birmingham,* *B15 2TT, UK.*

*^c^ Institute for Complexity Science, Henan University of Technology, Zhengzhou 450001, PR China.*

*^d^ Institute of Chemistry, Henan Academy of Sciences, Zhengzhou 450046, PR China.*

**Corresponding Authors: Lan Cui and Peng Li.*

*E-mail address:* [*cuilanmm56@163.com*](mailto:cuilanmm56@163.com) *(L. Cui),* lipeng@haut.edu.cn *(P. Li).*

**Fig.S1.** Surface morphology and crystallization property of Au NRs under different conditions. (A) TEM images of Au NRs synthesis via different NaBH_4_, HCl and AgNO_3_ concentrations. (B) Length-to-diameter ratio of Au NRs. (C) XRD analysis of Au NRs. (E-F) Photothermal conversion curves of Au NRs under 808 nm and 1064 nm laser over four laser irradiation on/off cycles (1 W/cm^2^, 5 min). Data are presented as mean ± standard deviation (SD, n = 3). Student’s t test, *p < 0.05, **p < 0.01, ***p < 0.001, ****p < 0.0001 indicate the statistically significant and extremely statistically significant, respectively.

**Fig.S2.** Cellular uptake and cell membrane integrity after 4 h treatment. (A) Fluorescent micrographs via confocal microscopy (scale bar:20 μm). (B) Fluorescence intensity analysis. (C) LDH related enzyme activity. Data are presented as mean ± standard deviation (SD, n = 3). Student’s t test, *p < 0.05, **p < 0.01, ***p < 0.001, ****p < 0.0001 indicate the statistically significant and extremely statistically significant, respectively.

**Fig. S3.** ROS quantification and mitochondrial membrane potential fluorescence quantification of Lipid 2-DG@Au/DOX-ss in MCF-7/ADR cells after 4 h. A) Fluorescent micrographs of ROS. B) Fluorescent micrographs of mitochondrial membrane potential via confocal microscopy. C) Fluorescence intensity analysis of ROS. D) Fluorescence intensity analysis of mitochondrial membrane potential. scale bar:20 μm. Data are presented as mean ± standard deviation (SD, *n* = 3). Student’s t test, *p < 0.05, **p < 0.01, ***p < 0.001, ****p < 0.0001 indicate the statistically significant and extremely statistically significant, respectively.

**Fig.S4.** Cell viability after Lipid 2-DG@Au/DOX-ss treatment at different 2-DG concentrations for 24 and 48 h. Data are presented as mean ± standard deviation (SD, *n* = 5). Student’s t test, *p < 0.05 indicates the statistically significant.

**Fig. S5.** Cell cycles and apoptosis analysis of Lipid 2-DG@Au/DOX-ss after 12 h. (A) Cell cycles. (B) Flow cytometry analysis of cell apoptosis after therapy. C) Cell cycles analysis. D) Cell apoptosis analysis. Data are presented as mean ± standard deviation (SD, n = 3). Student’s t test, *p < 0.05, **p < 0.01, ***p < 0.001, ****p < 0.0001 indicate the statistically significant and extremely statistically significant, respectively.
